# Gut microbiota-derived metabolites and IL-22 signaling in inflammatory bowel disease: context-dependent regulation of mucosal repair and inflammation

**DOI:** 10.3389/fimmu.2026.1891172

**Published:** 2026-08-25

**Authors:** Peixin Shi, Xiyuan He, Ling Zhao, Weisong Pan, Mingmei Zhou, Ting Zhang

**Affiliations:** 1 Institute of Interdisciplinary Integrative Medicine Research, Shanghai University of Traditional Chinese Medicine, Shanghai, China; 2 Wuhan Institute for Drug and Medical Device Control, Wuhan, China

**Keywords:** gut microbiota, IBD, IL-22, microbial metabolites, mucosal immunity

## Abstract

Inflammatory bowel disease (IBD), comprising Crohn’s disease (CD) and ulcerative colitis (UC), arises from complex interactions among host susceptibility, microbial dysbiosis, altered metabolite profiles, and dysregulated mucosal immune responses. Interleukin-22 (IL-22), a cytokine that acts primarily on non-hematopoietic cells, contributes to epithelial antimicrobial defense, survival, and tissue repair, but may also amplify inflammation under specific chronic or co-inflammatory conditions. This review evaluates the evidence linking major microbiota-associated metabolites—including short-chain fatty acids (SCFAs), bile acids (BAs), tryptophan (Trp) -derived metabolites, and the trimethylamine (TMA)/trimethylamine N-oxide (TMAO) pathway—to IL-22 production, bioavailability, and function in IBD. Relatively direct mechanistic support is available for SCFA-mediated and Trp-Aryl hydrocarbon receptor (AhR) -dependent regulation of IL-22, whereas bile acid effects appear metabolite-, receptor-, and context-dependent. In contrast, the proposed relationship between TMA/TMAO and IL-22 is currently supported mainly by indirect evidence and should be considered an emerging hypothesis. We further incorporate IL-22-binding protein (IL-22BP) as a determinant of local IL-22 bioavailability and distinguish upstream regulation of IL-22 production from downstream signaling initiated through the epithelial IL-22R1/IL-10R2 receptor complex. Particular attention is given to canonical JAK1/TYK2-STAT3 signaling and its context-dependent interactions with NF-κB, PI3K-AKT-mTOR, and MAPK pathways. We propose that the duration and magnitude of IL-22 exposure, its cellular source, the IL-22/IL-22BP balance, epithelial target-cell state, concurrent inflammatory signals, and disease context collectively determine whether IL-22 promotes mucosal repair or contributes to inflammatory amplification. By separating established mechanisms from associative, extrapolated, and hypothesis-generating evidence, this review provides a more evidence-calibrated framework for investigating the microbiota-metabolite-IL-22 network in UC and CD.

## Graphical Abstract

**Figure:**
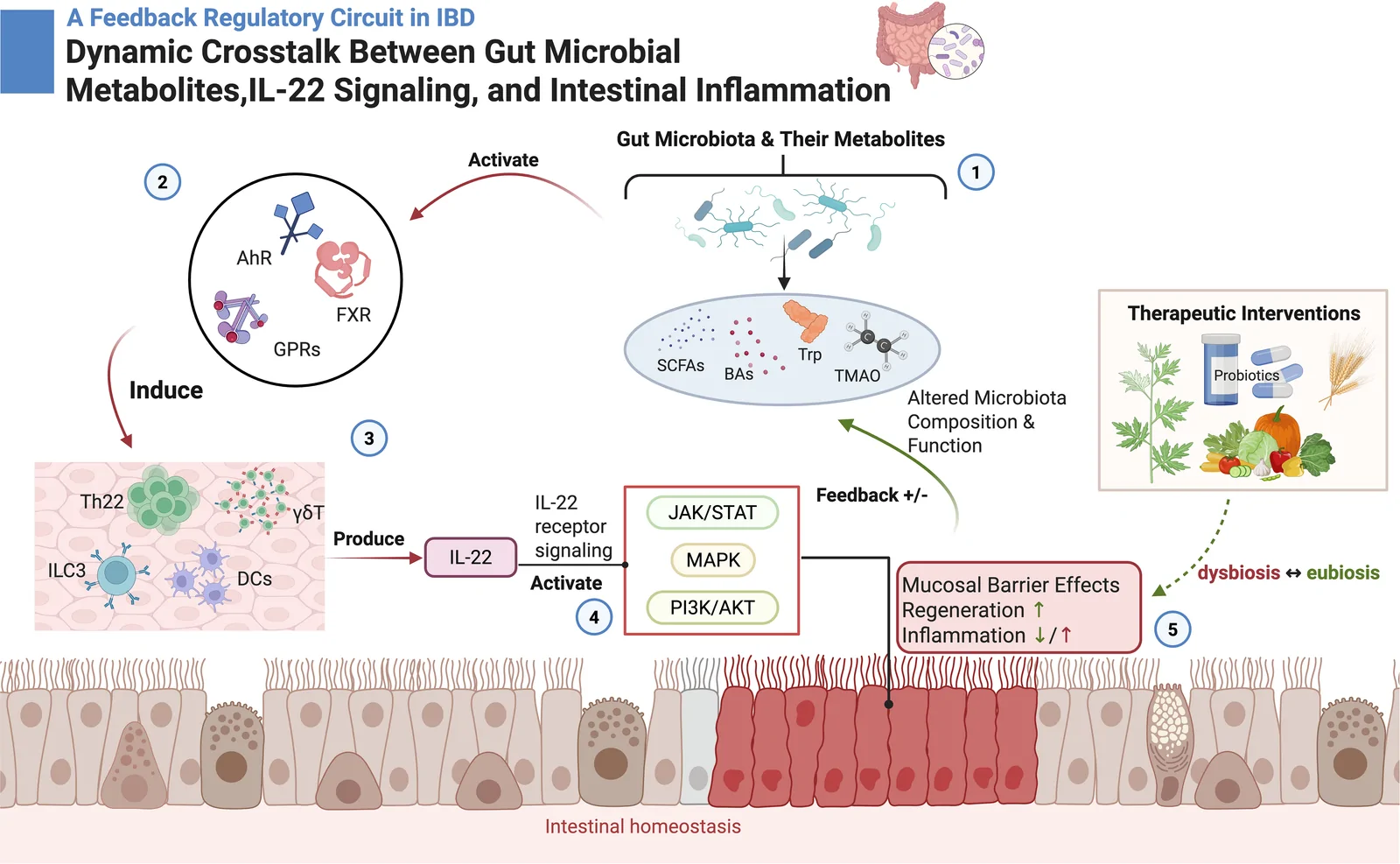
Dynamic crosstalk between gut microbial metabolites, IL-22 signaling, and intestinal inflammation: A feedback regulatory circuit in IBD. Overview of the feedback regulatory circuit involving dynamic crosstalk between gut microbial metabolites, IL-22 signaling, and intestinal inflammation, synthesizing the current understanding of this axis to provide insights into IBD pathogenesis and potential therapeutic strategies targeting the microbiota-metabolite-IL-22 signaling network for restoring intestinal homeostasis.

## Introduction

Inflammatory bowel disease (IBD) imposes a substantial and escalating global health burden, with its incidence rapidly rising, particularly in newly industrialized nations previously considered low-risk areas (1, 2). This group of chronic, relapsing inflammatory disorders targets the gastrointestinal tract, manifesting primarily as two distinct clinical phenotypes: ulcerative colitis (UC), characterized by continuous mucosal inflammation confined to the colon, and crohn’s disease (CD), which can affect any part of the gastrointestinal tract with transmural, discontinuous inflammation (3, 4). The etiology of IBD is multifactorial and reflects a confluence of host genetic predispositions, environmental factors, and perturbations of the gut microbiota, a condition known as dysbiosis (5–7). Clinically, patients often present with abdominal symptoms such as diarrhea, abdominal pain, bloody stools, and vomiting (8). These elements converge to disrupt intestinal immune homeostasis, ultimately leading to compromised epithelial barrier function and persistent inflammation (9–11). A central feature of IBD is a profound imbalance between pro-inflammatory and anti-inflammatory signals, which drives excessive immune cell infiltration and perpetuates tissue damage. In a significant subset of patients, this unrelenting inflammation creates a microenvironment that elevates the risk of developing colorectal cancer, a devastating long-term consequence of IBD (12). Consequently, therapeutic strategies for IBD have increasingly focused on neutralizing key cytokines to re-establish immune balance and mitigate these severe outcomes (13).

The gut microbiota, a dense and dynamic ecosystem residing in the gut lumen, is indispensable for shaping host immunity and maintaining mucosal health. This microbial community exerts its influence largely through a vast array of metabolic products, including short-chain fatty acids (SCFAs), modified bile acids (BAs), tryptophan (Trp) catabolites, and trimethylamine N-oxide (TMAO). These metabolites, which function as key signaling molecules, mediate the intricate communication between microbes and the host immune system. Consequently, disruptions in this metabolic signaling network are increasingly recognized as fundamental drivers in the initiation and progression of IBD (14–17).

Interleukin-22 (IL-22), a member of the IL-10 cytokine family, is produced primarily by innate lymphoid cells (ILCs) and selected T-cell subsets and acts mainly on non-hematopoietic target cells at the intersection of microbial signaling and mucosal immunity (18). IL-22 exhibits a well-documented dichotomous nature: it plays essential protective roles by promoting epithelial cell proliferation, stimulating antimicrobial peptide production, and enhancing barrier integrity, thereby facilitating tissue repair. However, under certain conditions, particularly in chronic inflammatory settings, IL-22 can also contribute to pathology, potentially by promoting neutrophil recruitment or driving aberrant cellular responses (19–27).

Gut microbiota-associated metabolites may influence IL-22 production through metabolite- and cell-specific mechanisms. Relatively direct evidence supports roles for SCFA sensing and histone deacetylase inhibition, as well as Trp-derived AhR ligands, whereas the effects of bile acid receptors and the TMA/TMAO pathway on IL-22 remain less completely defined (28–33). Furthermore, the ultimate biological consequences of IL-22 signaling depend on its engagement of downstream intracellular pathways, most notably the janus kinase/signal transducer and activator of transcription [JAK/STAT (primarily STAT3)], IκB kinase β (IKKβ) and nuclear factor kappa B (NF-κB), mechanistic target of rapamycin (mTOR), and mitogen-activated protein kinase (MAPK) cascades. The activation state and crosstalk between these pathways may be indirectly influenced by the metabolic milieu, dictating whether IL-22 signaling will achieve protective or detrimental outcomes in the inflamed intestine.

This review synthesizes the current understanding on the intricate relationship between gut microbial metabolites, IL-22 regulation, and the downstream signaling networks that govern intestinal inflammation and repair intestinal damage in IBD. By examining this regulatory network, we aim to provide novel insights into IBD pathogenesis and highlight potential therapeutic strategies targeting the microbiota-metabolite-IL-22 signaling network for restoring intestinal homeostasis.

Previous reviews have generally examined microbial metabolite alterations in IBD, IL-22 biology, or the Trp-AhR-IL-22 pathway as partially separate topics. The present review instead uses an integrative but evidence-calibrated framework that links metabolite identity and host sensing to IL-22-producing cell populations, IL-22BP-dependent cytokine availability, epithelial IL-22R1/IL-10R2 signaling, and context-dependent functional outcomes. Importantly, this framework does not assume that all proposed metabolite-IL-22 relationships are equally established. We distinguish direct mechanistic evidence from clinical or experimental associations, cross-model extrapolations, and emerging hypotheses. We then examine how the duration and magnitude of IL-22 exposure, its cellular source, target-cell state, concurrent inflammatory pathways, and differences between UC and CD may influence the balance between epithelial repair and inflammatory amplification. This approach is intended to define both the current evidence and the principal gaps that must be addressed before the microbiota-metabolite-IL-22 network can be translated into therapeutic strategies.

## Literature search strategy and evidence appraisal

This narrative review was informed by a structured literature search of PubMed and Web of Science databases from database inception to July 2026. Search terms were used individually and in Boolean combinations and included “inflammatory bowel disease,” “Crohn’s disease,” “ulcerative colitis,” “interleukin-22,” “IL-22,” “IL-22-binding protein,” “IL-22BP,” “gut microbiota,” “microbial metabolites,” “short-chain fatty acids,” “butyrate,” “bile acids,” “tryptophan metabolites,” “indole,” “aryl hydrocarbon receptor,” “trimethylamine,” “TMA,” “trimethylamine N-oxide,” “TMAO,” “JAK/STAT,” “NF-κB,” “mTOR,” and “MAPK.” Reference lists of relevant original studies and reviews were also screened to identify additional publications.

Peer-reviewed English-language studies were considered if they provided human, animal, organoid, cellular, or mechanistic evidence relevant to microbial metabolites, IL-22 production or bioavailability, IL-22 receptor signaling, epithelial responses, or IBD pathogenesis. Studies were excluded when they were duplicates, were not relevant to intestinal or mucosal biology, or did not provide sufficient evidence to support the claim for which they were considered. Evidence from non-intestinal tissues, non-IBD disease models, or non-mammalian organisms was included only when it offered potentially relevant mechanistic context and is explicitly identified as extrapolative.

To improve interpretive transparency, the included evidence was qualitatively classified as direct mechanistic, associative, extrapolated, or hypothesis-generating. Direct mechanistic evidence required experimental manipulation of a metabolite, receptor, cytokine, or signaling component together with measurement of an IL-22-related outcome. Associative evidence included clinical correlations or observational experimental findings without direct causal testing. Extrapolated evidence was derived from non-intestinal tissues, non-IBD models, or non-mammalian systems. Hypothesis-generating relationships were biologically plausible but lacked direct experimental validation. Human IBD evidence was prioritized where available, followed by interventional animal studies and mechanistic cellular studies. Conflicting results were retained and discussed rather than selectively excluded. Because this article is a narrative review, no formal risk-of-bias assessment or systematic-review protocol was applied.

## The pathogenesis of inflammatory bowel disease

IBD, primarily comprising CD and UC, manifests as chronic, relapsing inflammation of the gastrointestinal tract. A breakdown in immune homeostasis is central to IBD pathophysiology, characterized by skewed pro-versus anti-inflammatory cytokine profiles, excessive leukocyte infiltration into the intestinal lamina propria, and aberrant intracellular signaling (34). The commensal intestinal microbiota is integral to maintaining mucosal homeostasis, exerting considerable influence over both innate and adaptive immunity (14). Consequently, the dysbiosis frequently observed in IBD patients, marked by shifts in microbial community structure and function, is strongly implicated in driving persistent inflammation, often linked to altered microbial metabolite production and subsequent modulation of host signaling pathways. Within this complex immune network, various cell types contribute to the inflammatory milieu and tissue response. During acute inflammation, innate immune cells such as macrophages and neutrophils release reactive oxygen species (ROS) and inflammatory mediators. In parallel, group 3 innate lymphoid cells (ILC3s) secrete IL-22 to promote epithelial repair. In the adaptive immune response, T helper 17 (Th17) cells produce IL-17A and IL-22, while Th22 cells specialize in IL-22 production. Dysregulated activity of these cell populations in IBD contributes to chronic inflammation (35, 36). Among the cytokines involved in mucosal injury and repair, IL-22 is of particular relevance because it links immune-cell activation to epithelial antimicrobial, regenerative, and inflammatory programs (18).

## Intestinal microbiota-derived metabolites as immune modulators

The human gastrointestinal tract contains a dense and metabolically active microbial community. The collective metabolic activities of the microbiota generating a diverse pool of bioactive molecules that can influence host physiology. Beyond their contributions to host energy metabolism, these microbiota-derived metabolites are increasingly recognized as mediators in the establishment and maintenance of host immune homeostasis (37–40). Certain classes of metabolites, including SCFAs, Trp catabolic derivatives, and BAs have emerged as key modulators of host immunity. Disruptions in the production or signaling of these metabolites are strongly implicated in the pathogenesis of IBD (15–17). These molecules exert their effects by binding to specific host receptors, thereby influencing downstream signaling pathways and shaping the intestinal immune environment.

## IL-22 production, bioavailability, and functions in intestinal homeostasis

IL-22 is predominantly secreted by distinct immune cell populations, including ILC3s and adaptive T cell subsets such as Th17, Th22, and gamma delta (γδ) T cells. IL-22 signals through a heterodimeric IL-22R1/IL-10R2 receptor complex. The restricted expression of IL-22R1 largely determines cellular responsiveness to IL-22, whereas IL-10R2 is more broadly expressed. In the intestine, IL-22R1 is expressed predominantly by non-hematopoietic cells, particularly intestinal epithelial cells. Consequently, IL-22 primarily translates immune-cell activation into epithelial antimicrobial, survival, metabolic, and regenerative responses rather than acting directly on most immune cells (22). Consequently, IL-22 exerts its biological functions mainly by modulating the behavior of these stromal and epithelial cells rather than directly targeting immune cells. This targeted action underpins the well-established role of IL-22 in promoting tissue protection, repair, and antimicrobial defense.

In the intestine, IL-22 signaling enhances epithelial barrier integrity, induces the production of antimicrobial peptides, stimulates epithelial cell proliferation and survival, and promotes the regeneration of intestinal stem cells (e.g., LGR5+ cells) following injury. Specifically, the activation of STAT3 transcription factors mediated by IL-22 in intestinal epithelial cells is a critical pathway driving mucosal regeneration and repair. Furthermore, IL-22 contributes to coordinate the endoplasmic reticulum (ER) stress response in colonic epithelial cells, which may have context-dependent effects in CD (23).

**Figure 1:**
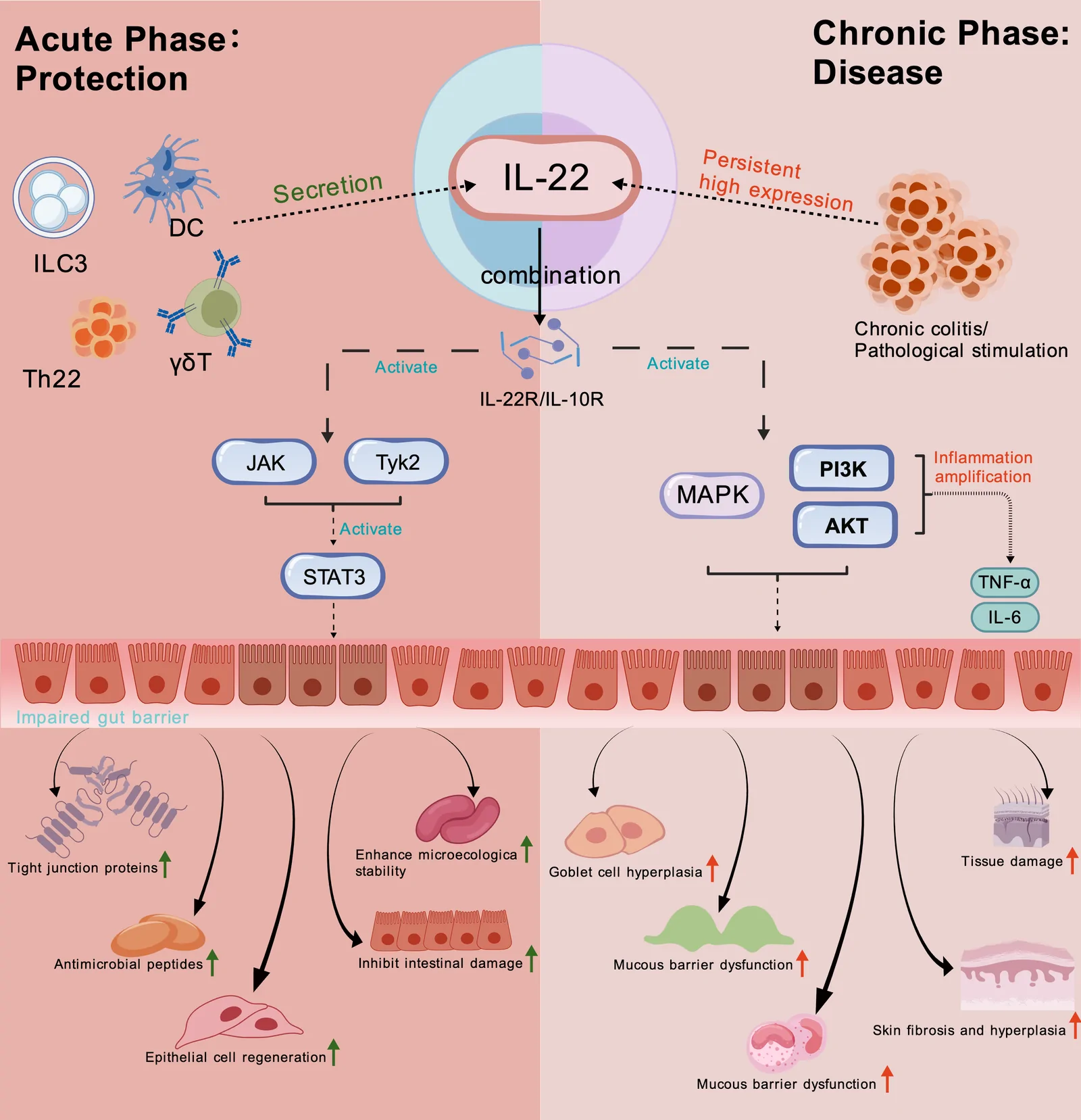
The dichotomous role of IL-22 in the pathogenesis of IBD. Schematic illustration of the dual functionality of IL-22 in IBD pathogenesis, depicting its protective roles in promoting tissue repair and barrier function during acute injury, contrasted with its potential to exacerbate inflammation in chronic settings.

However, the role of IL-22 in IBD pathogenesis is complex and dichotomous. While beneficial in acute injury settings, sustained or excessive IL-22 signaling in the context of chronic inflammation may paradoxically drive pathology (26, 27). This dual functionality is visually summarized in **Figure 1**, positioning IL-22 as a critical, context-dependent regulator in IBD pathogenesis.

### IL-22BP regulates IL-22 bioavailability

The biological consequences of IL-22BP are context-dependent (41). During acute epithelial injury, excessive neutralization of IL-22 may restrict antimicrobial defense, stem-cell regeneration, or wound repair (42). Conversely, insufficient control of sustained IL-22 activity may permit prolonged epithelial proliferation or inflammatory chemokine production. Human and experimental studies indicate that IL-22BP regulation is relevant to intestinal inflammation, but its cellular sources, temporal dynamics, and disease-specific functions remain incompletely resolved (43, 44). Moreover, direct evidence that individual microbial metabolites regulate IL-22BP in IBD is currently limited. Metabolite-dependent regulation of the IL-22/IL-22BP balance should therefore be regarded as an important research question rather than an established component of every metabolite-IL-22 pathway.

### Determinants of protective versus pathogenic IL-22 activity

The transition between protective and pathogenic IL-22 activity is unlikely to be controlled by a single variable (45). During acute or self-limited injury, transient IL-22 signaling generally supports antimicrobial peptide production, epithelial survival, and tissue regeneration (25, 27, 46, 47). In contrast, prolonged or excessive IL-22 exposure in chronically inflamed tissue may cooperate with other cytokine and stress pathways to amplify chemokine expression, neutrophil recruitment, or abnormal epithelial proliferation.

Several interacting factors may determine the net outcome. These include the duration and magnitude of IL-22 exposure; the cellular source of IL-22; the local IL-22/IL-22BP balance; the differentiation, metabolic, and stress state of IL-22-responsive epithelial cells; and concurrent activation of pathways driven by IL-23, IL-6, TNF, IL-1β, IL-18, STAT1, NF-κB, or MAPKs (5). Anatomical location, disease phase, and differences between UC and CD may further modify these responses. Thus, IL-22 concentration alone is unlikely to serve as a sufficient indicator of biological activity. Assessment of IL-22 should ideally be combined with measurements of IL-22BP, receptor expression, epithelial pathway activation, and disease context (18, 23, 48).

## Microbial metabolites and regulation of IL-22

**Figure 2:**
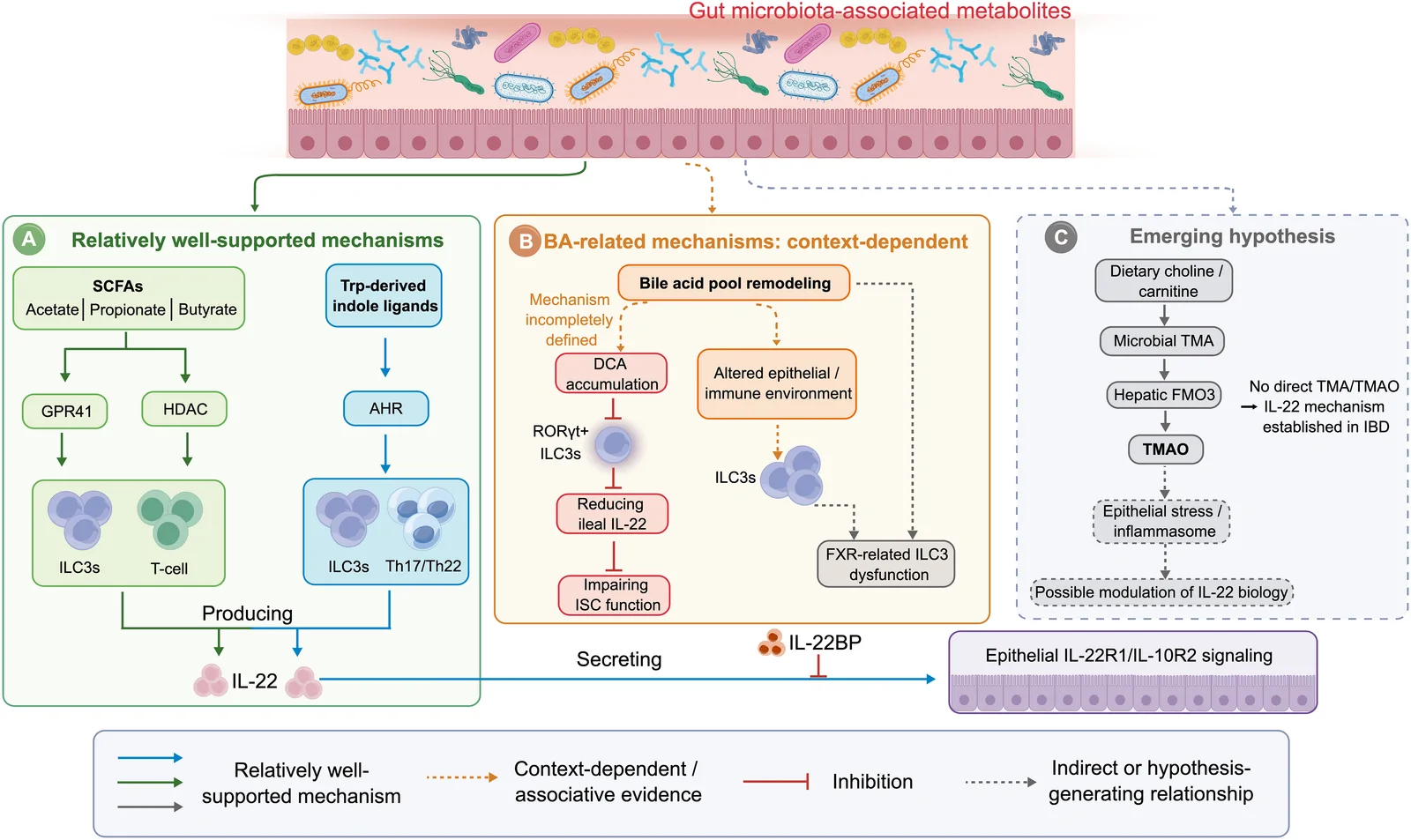
Evidence-calibrated upstream regulation of IL-22 production and bioavailability by microbiota-associated metabolites. **(A)** SCFAs and Trp-derived indole ligands regulate IL-22 production through relatively well-supported mechanisms involving SCFA sensing/HDAC inhibition and AhR activation, respectively. **(B)** Bile acid-related effects are metabolite- and context-dependent. **(C)** The proposed TMA/TMAO–IL-22 relationship remains hypothesis-generating. IL-22BP limits the amount of IL-22 available for epithelial IL-22R1/IL-10R2 signaling.

The intestinal microbiota is integral to host physiology, and its influence is largely mediated by the production of various metabolites that modulate immune homeostasis and gut health. Gut microbial metabolites exert profound regulatory control over IL-22 production, thereby shaping the intestinal immune environment. The gut microbiota metabolizes dietary components and host-derived substrates, such as undigested carbohydrates and amino acids, to generate a vast array of bioactive molecules. These metabolites directly act on host cells to promote or inhibit the secretion of IL-22 by key immune cell populations. This section dissects how distinct classes of metabolites, including SCFAs, BAs, Trp metabolites and their derivatives, and TMAO, orchestrate the complex regulation of IL-22. A comprehensive overview of how these different metabolites modulate IL-22 expression through specific molecular mechanisms is depicted in **Figure 2** and **Table 1**.

**Table 1:** Evidence summary of microbiota-associated metabolites in the regulation of IL-22 biology in IBD.

Metabolite	Proposed sensor and target cell	Experimental model	Disease/tissue context	Human IBD evidence	Direction of IL-22-related effect	Evidence strength	References
SCFAs	GPR41/HDAC-related mechanisms; ILC3s, CD4+ T cells, and other IL-22-producing lymphocytes	Murine immune-cell and colitis models; Human CD4+ T-cell experimentsILC3s	Intestinal immune regulation and experimental colitis	Evidence for SCFA-responsive IL-22 production in human CD4+ T cells; limited phenotype-stratified UC/CD data	Generally increases IL-22 production through receptor-dependent and epigenetic mechanisms	Relatively strong	(51–53)
Individual BAs and GA-associated BA remodeling	ILC3s, receptor and BA species responsible remain unresolved; Putative ILC3-IL-22-ISC axis, RORγt+ ILC3s and ISCs; Th17/Treg-associated cytokine environment	Murine colitis and metabolomic studies; High-fat-diet mouse model; TNBS-induced colitis model	Experimental colitis; Terminal ileum, epithelial repair and stem-cell function	No direct human IL-22 evidence	Changes in BA profiles are associated with increased ILC3 abundance and IL-22, direction varies by BA species and inflammatory setting; Associated with reduced ileal IL-22, reduced RORγt+ ILC3s, and impaired ISC proliferation/differentiation; Improves colitis while decreasing IL-22 and other inflammatory cytokines	Moderate	(31, 62–66)
Trp-derived indoles	AhR; primarily ILC3s and selected T-cell subsets, followed by epithelial IL-22R1/IL-10R2 signaling	Murine colitis models, pig model, and mechanistic immune-cell studies	Intestinal mucosal defense and experimental colitis	Altered Trp metabolism is reported in IBD; direct matched metabolite-IL-22 human studies remain limited	Indole-type AhR ligands generally increase IL-22 production and epithelial antimicrobial/repair responses	Relatively strong	(80–82, 86–88, 94)
TMA/TMAO	Intestinal epithelial cells, indirect effects on Th17/ILC3 cytokine environment; Myeloid and epithelial stress pathways, NLRP3, autophagy, ER stress, and mitochondrial dysfunction	Caco-2 cells and experimental intestinal-injury models; Cardiovascular, colorectal cancer, and non-intestinal inflammatory models, selected intestinal models	Epithelial stress and intestinal inflammation; Primarily non-IBD or non-intestinal contexts	No direct human IBD evidence linking TMA to IL-22; Altered TMAO has been reported in IBD, but no longitudinal paired TMAO-IL-22/IL-22BP studies are available	Increases IL-6/IL-1β and epithelial stress; may indirectly modify IL-22 production; May alter the inflammatory environment or epithelial responsiveness to IL-22, direct regulation of IL-22 has not been demonstrated	Hypothesis	(95–97, 99, 101, 103–105)

Evidence strength was assessed qualitatively. “Relatively strong” indicates direct experimental manipulation of a metabolite or relevant sensor together with an IL-22-related outcome in intestinal or immune-cell systems. “Moderate” indicates supportive but incomplete mechanistic evidence. “Hypothesis” indicates biologically plausible relationships lacking direct validation.

### SCFAs and regulation of IL-22

SCFAs are organic acids containing fewer than six carbon atoms and represent the most important metabolites produced by the human gut microbiota. The principal colonic SCFAs are acetate, propionate, and butyrate, which are generated mainly through microbial fermentation of indigestible carbohydrates (49). SCFAs are primarily produced through the fermentation of indigestible dietary carbohydrates (mainly fiber) by anaerobic bacteria in the colon. Especially, butyrate is the primary energy substrate for colonic epithelial cells, directly contributing to the maintenance of intestinal barrier function (50). SCFAs constitute an important component of microbiota-host communication. A key aspect of this interaction involves the enhancement of IL-22, a vital cytokine for epithelial barrier defense and repair.

Although specific lymphocyte populations, including ILC3s and various T cell subsets, the classical sources of IL-22, SCFAs have been identified as potent inducers of IL-22 production in these cells (18, 30). SCFAs promote IL-22 production through at least two reported mechanisms: activation of G protein-coupled receptor 41 (GPR41) and inhibition of histone deacetylase (HDAC) activity. As potent signaling molecules, SCFAs act as ligands for GPR41, which is highly expressed on the surface of both CD4+ T cells and ILCs. Upon binding, GPR41 triggers a robust intracellular signaling cascade characterized by the phosphorylation of the MAPK pathway, specifically extracellular signal-regulated kinase 1/2 (ERK1/2) (51). This kinase activity is pivotal for the metabolic reprogramming of these immune cells; it facilitates the activation of the transcription factor hypoxia-inducible factor 1-alpha (HIF-1α), which directly binds to the IL-22 promoter to initiate transcription. Furthermore, GPR41 signaling may indirectly bolster the AhR pathway by enhancing the cellular metabolic fitness required for sustained cytokine production, thereby establishing GPR41 as a critical bridge between the gut metabolome and epithelial barrier integrity (52).

SCFAs exert a profound influence on the transcriptional program of IL-22-producing cells by acting as endogenous HDAC inhibitors. Butyrate and propionate, in particular, penetrate the plasma membrane to suppress HDAC activity, leading to a generalized increase in the acetylation of histone H3 at the IL-22 locus. This modification relaxes the chromatin architecture at the IL-22 and AhR loci, thereby increasing the recruitment of RORγt and AhR to their respective binding sites. HDAC inhibition by SCFAs has been shown to upregulate the expression of AhR and hypoxia-inducible factor 1α (HIF1α) themselves. These epigenetic changes may increase the accessibility of transcriptional programs associated with IL-22 production. Notably, this epigenetic remodeling is not merely auxiliary; rather, it serves to lower the activation threshold for T cells and ILCs, ensuring a rapid and potent IL-22 response during intestinal dysbiosis or inflammatory challenge (30, 53).

Downstream of these initial signals, SCFAs enhance the expression and/or activity of transcription factors pivotal for IL-22 gene expression, including the AhR and HIF1α. Functionally, SCFA supplementation in experimental models elevates intestinal IL-22 levels and confers protection against inflammatory challenges. Importantly, SCFAs have also been shown to stimulate IL-22 production from human CD4+ T cells, highlighting the translational relevance of this regulatory axis.

Intriguingly, evidence from non-mammalian models suggest that SCFA-mediated IL-22 induction extends beyond conventional lymphoid cells. A zebrafish study reported butyrate-induced IL-22 expression in fish macrophages. This finding provides comparative evidence that SCFA-responsive IL-22 production may extend beyond conventional lymphoid populations in some species; however, it does not establish macrophages as a relevant source of IL-22 in human IBD and should not be directly extrapolated to mammalian intestinal immunity (54).

Collectively, SCFAs as key microbial metabolites directly stimulate IL-22 production from critical innate and adaptive immune cells, thereby reinforcing intestinal barrier function and promoting immune homeostasis. The SCFA-IL-22 relationship is supported by mechanistic evidence in experimental models and selected human immune-cell studies. Nevertheless, its therapeutic translation will require definition of metabolite dose, delivery, cell-specific responses, and disease-stage dependence.

### BAs and regulation of IL-22

BAs are steroid acids, primarily biosynthesized from cholesterol in the liver via enzymes such as cholesterol 7α-hydroxylase (CYP7A1). BAs are essential for dietary lipid emulsification and absorption in the small intestine, and also act as signaling molecules that mediate complex interactions between the host and the intestinal microbiota, thereby significantly influencing mucosal immunity and epithelial cell function. BAs are generally divided into primary BAs and secondary BAs. Primary BAs, including cholic acid (CA) and chenodeoxycholic acid (CDCA), are typically conjugated with glycine or taurine before being secreted via bile into the duodenum. In the intestinal lumen, particularly the distal ileum and colon, conjugated primary BAs undergo extensive biotransformation by the resident microbiota. The key modifications, including deconjugation (removal of glycine/taurine) and 7α/β-dehydroxylation, are primarily catalyzed by enzymes expressed by anaerobic bacterial genera such as *Clostridium*, *Eubacterium*, and *Bacteroides*. These microbial activities generate a diverse pool of secondary BAs, including deoxycholic acid (DCA) and lithocholic acid (LCA). Crucially, secondary BAs often possess distinct physicochemical properties, receptor affinities, and biological activities compared to their primary precursors, underscoring the importance of the microbiota in shaping the functional BAs pool in the gut.

BAs exert potent immunomodulatory effects by acting as ligands for specific host receptors. Two major BAs receptors are the nuclear receptor farnesoid X receptor (FXR) and the membrane-bound takeda G protein-coupled receptor 5 (TGR5). FXR, highly expressed in hepatocytes and intestinal epithelial cells, is preferentially activated by conjugated and unconjugated primary BAs, particularly CDCA. Activation of epithelial FXR is generally associated with enhancing intestinal barrier integrity and suppressing pro-inflammatory gene expression, thereby contributing to the maintenance of immune tolerance (55). In contrast, TGR5 is expressed on various cell types, including intestinal epithelial cells (especially L cells), macrophages, and dendritic cells. TGR5 exhibits higher affinity for certain secondary BAs, notably LCA and DCA (56–59). TGR5 signaling has been implicated in diverse functions, including the release of incretin hormones (like GLP-1) from L cells and the modulation of inflammatory responses in immune cells, often promoting anti-inflammatory effects, such as potentially facilitating the release of IL-10 in certain contexts (60). The metabolic activities of the gut microbiota determine the relative abundance of primary and secondary BAs, critically shaping host immune homeostasis through differential engagement of these receptors.

BAs can influence IL-22 production, but their effects appear to be bidirectional and highly context-dependent. Compared with SCFAs, much of the evidence linking BAs to IL-22 remains associative rather than directly mechanistic. This heterogeneity is biologically plausible because BA activity varies according to the identity and concentration of individual BA species, receptor affinity, target-cell type, intestinal location, and inflammatory milieu. Microbiota-dependent transformation of BAs may also contribute to IBD pathogenesis by reshaping immune-cell differentiation, including the balance between regulatory T cell (Treg) and Th17 cells, an important component of intestinal immune regulation (37). Within this setting, IL-22 supports epithelial integrity and wound healing and has also been implicated in Paneth cell metaplasia in the colonic epithelium and *REG3A*-associated mucosal repair (61).

Certain secondary BAs may impair IL-22 production and its associated tissue-protective functions. In a high-fat diet model, increased DCA was associated with impaired proliferation and differentiation of intestinal stem cells (ISCs), together with reduced ileal IL-22. Immunofluorescence analysis of the terminal ileum further showed a decrease in RORγt-expressing ILC3s, suggesting that loss or dysfunction of IL-22-producing cells may contribute to the observed ISC phenotype. These findings support a link among DCA accumulation, diminished ILC3-derived IL-22, and defective ISC activity; however, they do not yet establish that DCA directly exerts cytotoxic effects on ILC3s. Several, potentially overlapping processes may be involved, including epithelial or stem-cell stress, disruption of the epithelial niche, altered ILC3 survival or function, and secondary changes in the inflammatory environment. The temporal order and molecular intermediates connecting DCA exposure to reduced IL-22 and ISC dysfunction therefore remain unresolved (62). Additional evidence suggests that microbial remodeling of the BA pool may mitigate the intestinal toxicity associated with secondary BAs. A recent study showed that *Lactiplantibacillus plantarum* GR-4 promoted secondary BA sulfation, reduced BA-associated intestinal toxicity, and limited the accumulation of primary BAs during colitis. This intervention alleviated colitis through several coordinated mechanisms, including restoration of sulfolithocholic acid derivatives and correction of colitis-associated metabolic abnormalities, such as increased 7-sulfocholic acid (63). Although this study did not fully define whether IL-22 was required for the therapeutic effect, it provides a mechanistic basis for examining how microbial BA detoxification may preserve the epithelial and immune-cell environments needed for IL-22-dependent repair.

Other studies have linked individual BA species to intestinal IL-22 expression. In one murine study, ileal IL-22 expression was positively correlated with CA, with additional associations reported for taurocholic acid (TCA), glycocholic acid (GCA), CDCA, DCA, ursodeoxycholic acid (UDCA), tauroursodeoxycholic acid (TUDCA), and LCA. However, IL-22 expression increased alongside inflammatory mediators, including IL-1α, IL-1β, IL-6, IL-17A, TNF-α, and IFN-γ (64). Thus, the association may reflect a compensatory repair response accompanying intestinal inflammation rather than direct induction of IL-22 by each BA species. This distinction is important because an increase in IL-22 during active inflammation cannot, by itself, establish whether IL-22 is limiting tissue injury or participating in inflammatory amplification. A recent study examining gallic acid (GA) treatment provided further evidence that microbiota-dependent BA remodeling may influence ILC3 responses. GA altered gut microbial composition and BA metabolism, increased the proportion of ILC3s, and raised concentrations of anti-inflammatory or tissue-protective mediators, including IL-22. Both primary and secondary BAs were associated with improvement of experimental colitis, while LCA, isoalloLCA, 3-oxoLCA, UDCA, and CDCA correlated positively with bacterial taxa considered beneficial in that model (65). These findings connect BA remodeling with ILC3 abundance and IL-22 production, but they do not yet identify which BA species acts directly on ILC3s, which receptor mediates the response, or whether the increase in IL-22 is secondary to broader restoration of the intestinal microbial environment.

The relationship between BA metabolism and IL-22 is not uniformly positive. Taurohyodeoxycholic acid (THDCA), for example, alleviated TNBS-induced colitis while reducing several inflammatory cytokines, including IL-6, IL-17A, IL-21, IL-22, and TNF-α, and increasing TGF-β1 and IL-10 (66). This observation illustrates why a reduction in IL-22 should not automatically be interpreted as loss of mucosal protection. In this model, lower IL-22 may instead reflect attenuation of the inflammatory stimuli that drive its compensatory or pathogenic production. The net effect of a BA intervention must therefore be interpreted in relation to disease stage, the broader cytokine environment, and the cellular source of IL-22. Microbiota-directed interventions provide another route through which BA metabolism and IL-22 may intersect. Probiotic administration has been reported to alter both microbial composition and the BA profile, accompanied by changes in IL-22 abundance (67).

Dietary context may further modify this relationship. Fiber deficiency has been associated with an increased frequency of pro-inflammatory Th17 cells, potentially through dysbiosis and accompanying changes in BA composition. In contrast, genus *Alistipes* exerted protective effects in experimental colitis in association with increased colonic IL-22 and *Reg3γ* production (68). Whether BA remodeling is the intermediate step connecting this bacterium to the IL-22-*Reg3γ* response has not yet been resolved. Mechanistic studies combining microbial colonization, targeted BA profiling, receptor perturbation, and IL-22 blockade will be needed to distinguish BA-dependent effects from other microbial functions. Evidence from neonatal necrotizing enterocolitis (NEC) also indicates that FXR signaling can connect the BA environment to ILC3 function. In this model, FXR activation promoted ferroptosis in intestinal epithelial cells, impaired ILC3 proliferation, and reduced ILC3-derived IL-22. Conversely, the intestine-restricted FXR antagonist glycine-β-muricholic acid (Gly-β-MCA) showed therapeutic activity in mice (31). These findings provide a more defined BA-FXR-epithelial injury-ILC3/IL-22 pathway, but their relevance to adult UC and CD requires careful validation because NEC has a distinct developmental, microbial, and inflammatory context. Similar associations have been reported in intervention models of colonic inflammation. Cyanidin-3-O-glucoside (C3G) reversed the reduction in IL-22 expression induced by polystyrene exposure, enriched microbial genes involved in BA metabolism, and increased several related metabolites, including 3β-hydroxy-5-cholenoic acid, CDCA, taurine, and LCA (69). Although these concurrent changes suggest a connection between BA remodeling and restoration of IL-22, the study did not establish whether any individual metabolite directly induced IL-22 or whether both responses resulted from broader improvement of the intestinal environment. Curcumin has likewise been reported to remodel intestinal microbial communities and BA metabolism. In an experimental model, curcumin modulated the BA-FXR pathway, altered primary and secondary BA concentrations, including CDCA and LCA, and increased IL-22 secretion by lamina propria ILC3s, with accompanying improvement in intestinal inflammation and mucosal immune homeostasis (70). Nevertheless, the intervening molecular steps were not fully delineated. In particular, it remains unclear whether the change in ILC3-derived IL-22 resulted from direct BA receptor signaling in ILC3s, indirect effects of FXR signaling in epithelial or myeloid cells, or broader microbiota-dependent changes.

Taken together, current evidence supports a close but heterogeneous relationship between the intestinal BA pool and IL-22 biology. DCA accumulation may impair the ILC3-IL-22-ISC repair axis, whereas microbial or pharmacological remodeling of BA metabolism can coincide with recovery of ILC3 abundance, IL-22 production, and epithelial homeostasis. At the same time, some BA interventions alleviate colitis while lowering IL-22, indicating that the direction of IL-22 change cannot be interpreted independently of the inflammatory setting. BA receptors, including FXR and TGR5, regulate epithelial and innate immune functions, but direct evidence that their activation consistently increases IL-22 production in IBD remains limited. Future studies should identify the relevant BA species, receptor-expressing cells, and tissue compartments and should use receptor-specific perturbation and IL-22 or IL-22BP blockade to distinguish direct signaling from secondary effects of microbial and metabolic remodeling. These distinctions will be essential for evaluating microbiota- and receptor-directed strategies targeting the BA-IL-22 network in IBD.

### Trp metabolites and regulation of IL-22

Tryptophan (Trp), an essential amino acid for mammals, must be exogenously obtained from protein-rich foods such as poultry, fish, soybeans, quinoa, or endogenously produced through protein hydrolysis (71). Unabsorbed Trp is a crucial substrate for intestinal microbiota in the gastrointestinal tract. Trp metabolism proceeds via three major routes: the kynurenine pathway (KP), the serotonin (5-hydroxytryptamine, 5-HT) pathway, and the microbially driven indole pathway (72). Various bacterial genera, including members of *Lactobacillus* and *Bacteroides*, possess enzymes like tryptophanase that convert Trp into a diverse array of bioactive catabolites, such as indole and its derivatives indole-3-propionic acid (IPA), indole-3-acetic acid (IAA) and indole-3-aldehyde (IAld).

The KP accounts for the majority (~95%) of Trp catabolism in mammals, initiated by the rate-limiting enzymes indoleamine 2,3-dioxygenase (IDO1/IDO2) or tryptophan 2,3-dioxygenase (TDO). The downstream metabolites, including kynurenine (Kyn), 3-hydroxykynurenine (3-HK), and quinolinic acid (QA), exert immunomodulatory effects, potentially through AhR activation or direct effects on immune cells like inhibiting T cell proliferation (73). Kyn, for instance, contributes to immune tolerance by suppressing dendritic cell (DC) antigen presentation and promoting Treg differentiation (74). KP metabolites can influence IL-22 secretion by CD4+ T cells, thereby impacting intestinal barrier function. The KP possesses cytoprotective and immunomodulatory roles in various inflammatory conditions, including IBD (75). It is worth noting that the gut microbiota can influence Trp availability in the KP and directly metabolize Trp, which means dysbiosis can disrupt KP and exacerbate IBD inflammation (76, 77).

A smaller fraction of Trp (~1-2%) is converted to 5-HT by tryptophan hydroxylase (TPH), primarily in enterochromaffin cells and the central nervous system. A smaller proportion of Trp is metabolized through the serotonin pathway. Although serotonin can modulate intestinal immune and epithelial functions, its direct contribution to IL-22 regulation in IBD remains insufficiently defined (78).

The direct microbial metabolism of Trp via the indole pathway generates crucial AhR ligands. These molecules activate AhR in immune cells, particularly promoting IL-22 secretion by ILC3s and Th17 cells (15, 79). AhR activation induces the transcription factor RORγt in ILC3s and directly binds the IL-22 promoter, upregulating its expression (28). Subsequently, IL-22 acts on intestinal epithelial cells (IECs) via STAT3 signaling to stimulate epithelial cell proliferation, enhance barrier integrity, and induce antimicrobial peptide production, thereby strengthening mucosal defense (79, 80). The importance of this axis has been confirmed in studies showing reduced IL-22 levels and increased susceptibility to colitis in AhR deficient mice (81). Conversely, the interventions that enhance microbial production of indole type AhR ligands, such as supplementation with *Lactobacillus* or dietary pectin, can activate the AhR-IL-22 pathway and ameliorate experimental colitis (80, 82).

Microbiota-derived Trp catabolites function as pivotal bioactive cues that profoundly shape host immune homeostasis, a process largely orchestrated through the ligation of the AhR. As a quintessential ligand-activated transcription factor, AhR is ubiquitously expressed across diverse hematopoietic and non-hematopoietic lineages, thereby serving as a critical molecular sensor that translates microbial metabolic outputs into cell-specific transcriptional programs. Microbially derived indole metabolites can function as AhR ligands and promote IL-22 production, particularly in ILC3s and selected T-cell populations. The resulting IL-22 then acts on epithelial IL-22R1/IL-10R2 to activate predominantly STAT3-dependent programs. These programs enhance the expression of tight junction proteins, stimulate epithelial cell proliferation and repair, and promote mucus production, thus fortifying the intestinal barrier (28). This Trp-AhR-ILC-IL-22 axis represents a key pathway linking microbial metabolism to mucosal defense. In experimental colitis, *Akkermansia muciniphila* and its outer-membrane protein Amuc_1100 have been associated with altered Trp metabolism and enhanced AhR-related signaling. These findings suggest that microbiota-directed interventions may modify the availability of AhR ligands, although the extent to which this effect depends directly on IL-22 requires further mechanistic validation. Collectively, these experimental interventions suggest that microbiota-directed modulation of Trp metabolism may enhance AhR-IL-22-associated mucosal responses and improve experimental colitis; however, the relative contribution of individual microbial species, specific indole metabolites, and IL-22 itself requires further mechanistic clarification (83–88).

Furthermore, genetic predispositions affecting microbial sensing (e.g., CARD9 deficiency) can lead to dysbiosis, impaired Trp metabolism, reduced AhR signaling, deficient IL-22 production, and increased colitis susceptibility (89). AhR signaling may also balance IL-22’s functions by modulating Th17 responses, and different cell types exhibiting varying sensitivities (90). ILC3s are potentially more responsive to indole ligands through AhR, while Th17 regulation may involve KP metabolites (91).

Dysregulation of the Trp-AhR-IL-22 network is strongly implicated in IBD pathogenesis. In IBD patients, microbial dysbiosis can lead to changes in Trp metabolism, often characterized by reduced indole production and a shift towards the KP, resulting in insufficient AhR activation and decreased IL-22 levels, thereby impairing barrier function and sustaining inflammation (92). Human studies have reported heterogeneous associations between IL-22 abundance and IBD activity. IL-22 may reflect compensatory epithelial repair in some settings but may accompany inflammatory amplification in others; therefore, circulating or mucosal IL-22 levels should not be interpreted independently of disease type, tissue compartment, IL-22BP abundance, and downstream pathway activation (47, 93, 94). Although IL-22 has generally a protective effect in the gut, its role may depend on the environment.

In conclusion, Trp metabolism, particularly the microbial driven indole pathway via AhR, is a critical regulator of IL-22 production in the intestine. This network represents a key interface between the host, its microbiota, and mucosal immunity. The dynamic regulation of IL-22 by different Trp metabolic pathways and the crosstalk between these metabolites and IL-22 signaling remains an important area of future research, providing potential for novel therapeutic strategies for IBD and other immune-mediated diseases.

### TMA/TMAO and regulation of IL-22

The formation of trimethylamine (TMA) and its oxidation product, TMAO, depends on the coordinated activities of the intestinal microbiota and host metabolic enzymes. Metagenomic studies have identified several taxa with the capacity to produce TMA, including members of *Clostridium*, *Lachnoclostridium*, and *Anaerococcus*, as well as *Emergencia timonensis* and selected members of the *Ruminococcaceae* and *Lachnospiraceae* families (95–97). Dietary quaternary ammonium compounds, including choline, L-carnitine, phosphatidylcholine, and betaine, are converted to TMA by microbial enzymes in the intestine. Following absorption into the portal circulation, TMA is transported to the liver and oxidized to TMAO, predominantly by flavin-containing monooxygenase 3 (FMO3) (98). Host hepatic metabolism therefore represents an important determinant of circulating TMAO concentrations and completes the metabolic link among diet, the intestinal microbiota, and host physiology (99). *Fusobacterium* and *Desulfovibrio* species, together with methanogen *Methanobrevibacter smithii*, have also been associated with plasma TMAO concentrations (95). TMAO is thus more accurately regarded as a host-microbial co-metabolite whose abundance reflects microbial TMA-producing capacity as well as hepatic oxidation, renal clearance, and dietary exposure.

TMAO first attracted attention because of its association with cardiovascular disease(CVD). Multiple cohort studies and meta-analyses have reported positive relationships between circulating TMAO concentrations and myocardial infarction, stroke, and all-cause mortality (100–102). As understanding of microbiota-host metabolic interactions has advanced, interest has expanded beyond the use of TMAO as a circulating biomarker toward its possible contribution to diet-associated, microbial, and multiorgan inflammatory responses (95, 99). Evidence from intestinal models further suggests that TMA and TMAO may affect epithelial and mucosal biology, although the two metabolites should not be treated as mechanistically interchangeable. TMA exposure reduced transepithelial electrical resistance in Caco-2 monolayers, impaired mitochondrial ATP production, and altered mitochondrial DNA methylation (103, 104). TMAO, by comparison, has been reported to promote inflammation-associated colorectal tumorigenesis by binding the heat-shock cognate protein HSPA8 and stabilizing β-catenin (105). In a Psrc1-deficient mouse model, microbiota dysbiosis was accompanied by increased TMA production, higher circulating TMAO concentrations, and a pro-inflammatory colonic phenotype (106). Collectively, these observations extend the relevance of the TMA/TMAO pathway beyond systemic vascular injury and provide a rationale for investigating its contribution to intestinal mucosal inflammation (101, 107).

Although TMA/TMAO metabolism and IL-22 biology have each been examined in intestinal inflammation, a direct cross-regulatory relationship between them has not been established. Nevertheless, several lines of indirect evidence make a putative TMA/TMAO-IL-22 connection biologically plausible. First, TMAO can activate the NLRP3 inflammasome and interfere with autophagy-related pathways involving ATG16L1. In turn, ATG16L1 deficiency has been reported to amplify IL-22-induced type I interferon responses and aggravate ileal inflammation (108, 109). These findings do not demonstrate that TMAO directly regulates IL-22, but they identify autophagy and epithelial inflammatory signaling as potential points of convergence. Second, TMA can induce IL-6 and IL-1β expression in intestinal epithelial cells. Because the local cytokine environment contributes to the activation of IL-22-producing Th17 cells and ILC3s, TMA-induced inflammatory mediators could indirectly modify IL-22 production (103, 110). This possibility remains inferential, however, because a rise in IL-6 or IL-1β does not establish that TMA increases IL-22 secretion, and the outcome is likely to depend on the cellular composition and inflammatory state of the tissue. Third, several components of IL-22 biology, including AhR-dependent transcription, STAT3 activation, and stress-responsive transcriptional programs involving ATF3, are sensitive to cellular metabolism. TMAO-induced ER stress and mitochondrial dysfunction could therefore alter either the production of IL-22 or the response of epithelial cells to this cytokine (104, 105). The two possibilities should be distinguished: an effect on IL-22-producing immune cells would represent upstream regulation, whereas a change in epithelial stress, receptor competence, or STAT3-related signaling would modify the downstream consequences of IL-22 without necessarily changing its secretion. Clinical associations provide an additional, albeit non-causal, layer of support. In patients with IBD, circulating TMAO concentrations have been reported to show a nonlinear relationship with C-reactive protein, whereas IL-22 has been associated with inflammatory chemokines such as CCL24 (44, 111). These parallel associations raise the possibility that TMAO-related metabolism and IL-22 participate in overlapping inflammatory networks. They do not, however, demonstrate functional coupling between the two, because neither temporal sequence nor direct molecular interaction can be inferred from cross-sectional correlations. On this basis, the proposed TMA/TMAO-IL-22 relationship can be framed as a candidate regulatory module connecting microbial metabolism, epithelial stress, innate immune activation, and mucosal cytokine responses in IBD (18, 101, 103, 105, 107). The evidence supporting this model is multidimensional but remains indirect. No published study has yet demonstrated that TMA or TMAO acts directly on ILC3s or Th17 cells to regulate IL-22 transcription or secretion. IL-22 production is controlled by transcription factors such as AhR and RORγt and by cytokines including IL-23 and IL-1β, but whether TMA/TMAO modifies these upstream programs through metabolic reprogramming, epigenetic regulation, or RNA modifications such as N6-methyladenosine remains unknown (112). Current evidence is derived largely from clinical associations, parallel observations across experimental systems, or inference from adjacent pathways. In the absence of co-culture experiments, cell-specific receptor or enzyme perturbation, conditional genetic models, and single-cell or spatial analyses, a causal TMA/TMAO-IL-22 pathway cannot yet be defined.

Human evidence is similarly limited. The relationship between TMA/TMAO and IL-22 in IBD has been investigated mainly through cross-sectional observations and relatively small experimental studies. Large longitudinal cohorts stratified by disease phenotype—including UC versus CD, disease activity, anatomical location, and treatment exposure—remain unavailable. Although altered TMAO concentrations have been reported in IBD and mucosal IL-22 may be elevated and regulated by IL-22BP, it remains unclear whether these changes are temporally or functionally related during disease progression, therapeutic response, or relapse (41, 43). Importantly, the amount of IL-22 detected in tissue does not necessarily indicate its biological availability, because IL-22BP can restrict engagement of the epithelial IL-22R1/IL-10R2 receptor complex.

Dose and exposure duration represent further unresolved variables. No study has systematically compared the effects of different concentrations or durations of TMA/TMAO exposure on IL-22 and related Th17- or ILC3-associated cytokines, including IL-17A and IL-23. Evidence concerning the functional response of defined IL-22-producing cell populations is also lacking in IBD-relevant models such as IL-10-/- mice or T-cell transfer colitis (113). In particular, time-series studies are needed to determine whether changes in TMA or TMAO precede alterations in IL-22, occur as a consequence of inflammation-associated dysbiosis, or track with the activity of ILC3s, Th17 cells, and other mucosal immune populations (47, 114). Without this temporal information, the clinical validity and translational relevance of the proposed TMA/TMAO-IL-22 relationship remain uncertain. These gaps also have implications for patient stratification. Any future intervention targeting this pathway would need to account for IBD subtype, ileal versus colonic involvement, inflammatory activity, microbial TMA-producing capacity, hepatic FMO3 activity, and renal clearance. The therapeutic objective would not simply be to suppress TMAO or IL-22. Rather, it would be to reduce potentially pathogenic TMA/TMAO-associated inflammatory signals while preserving the epithelial repair functions of IL-22. Such a strategy will require biomarkers capable of distinguishing patients with metabolite-driven inflammatory amplification from those in whom insufficient IL-22 activity contributes to defective mucosal healing.

One potential intervention involves inhibiting microbial TMA formation. TMA lyase inhibitors target microbial enzymes that catalyze the conversion of choline or carnitine to TMA, including the CutC/CutD enzyme system. For example, 3,3-dimethyl-1-butanol (DMB) and iodomethylcholine (IMC) have reduced TMA production in animal models without causing broad disruption of overall microbial community structure (115). Their efficacy has not yet been directly evaluated in well-characterized IBD models. Nevertheless, because TMA can increase IL-6 and IL-1β expression, impair epithelial barrier function, disrupt mitochondrial metabolism, and promote colonic mucosal injury and inflammatory-cell infiltration, inhibition of microbial TMA production could theoretically reduce intestinal inflammatory pressure (103, 116). This remains a therapeutic hypothesis rather than evidence of efficacy in IBD. Newer inhibitors of microbial TMA formation may offer additional pharmacological options. R-N-fluoromethylcarnitine (FCAR), for example, has shown favorable pharmacokinetic characteristics and reduced blood and urinary TMA concentrations in experimental settings, without overt adverse effects such as weight loss (117). These findings support further preclinical evaluation, but they do not yet establish suitability for IBD treatment. Studies in colitis models will need to determine whether lowering TMA modifies mucosal inflammation, IL-22 production, IL-22BP abundance, epithelial signaling, and microbial ecology without introducing unintended metabolic effects. The therapeutic relationship with JAK-STAT signaling requires particular caution. IL-22 exerts many of its epithelial effects through JAK1/TYK2-dependent STAT3 phosphorylation, while JAK inhibition is already used therapeutically in IBD, as illustrated by tofacitinib and other agents (48, 110). At the same time, IL-22 can support barrier maintenance and tissue repair or contribute to pathological epithelial stress, depending on the inflammatory setting (23). Consequently, indiscriminate activation or inhibition of this pathway is unlikely to reproduce the context-specific balance required for mucosal recovery. Combined modulation of microbial TMA production and JAK-STAT signaling may therefore warrant investigation, but it should presently be regarded as a testable therapeutic concept. For example, a microbial TMA lyase inhibitor combined with carefully titrated JAK inhibition might reduce inflammatory amplification in a biomarker-defined subgroup characterized by excessive cytokine signaling and epithelial injury. This possibility would need to be compared with TMA-lowering treatment alone because even low-dose JAK inhibition could compromise IL-22-dependent epithelial repair. Conversely, in patients with defective mucosal regeneration, approaches that preserve or enhance appropriately localized IL-22 activity could be explored in organoid and preclinical systems (24, 118).

Overall, the TMA/TMAO-IL-22 model offers a useful framework for connecting microbial metabolism with epithelial stress, innate immune activation, and cytokine-dependent mucosal responses. Its value at present lies in generating experimentally testable questions rather than defining an established signaling pathway. Direct studies should determine whether TMA or TMAO alters IL-22 production by specific immune-cell populations, whether it modifies IL-22BP or epithelial IL-22 receptor signaling, and whether these interactions differ between UC and CD. Until such evidence becomes available, therapeutic proposals targeting this relationship should remain explicitly exploratory.

## Downstream IL-22 receptor signaling and context-dependent pathway crosstalk

**Figure 3:**
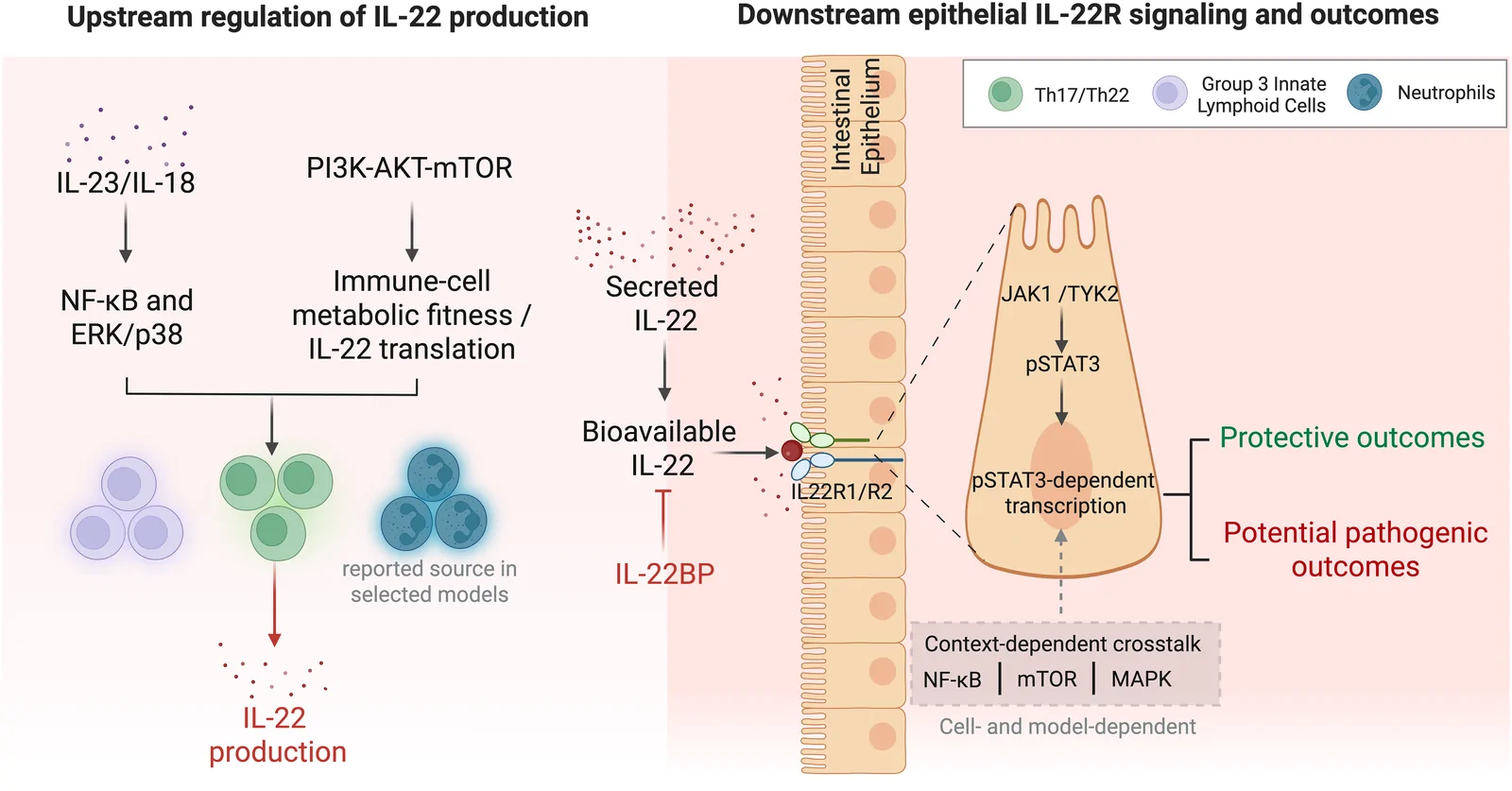
Upstream regulation of IL-22 production and downstream epithelial signaling in IBD. On the left, immune-cell pathways that regulate IL-22 production are shown, together with the IL-22BP-mediated restriction of secreted IL-22 availability for receptor engagement. On the right, canonical IL-22R1/IL-10R2–JAK1/TYK2–STAT3 signaling in epithelial cells is illustrated. NF-κB, mTOR, and MAPK are shown as context-dependent interacting pathways rather than equivalent direct outputs of IL-22R. The resulting epithelial response ranges from barrier repair to inflammatory amplification according to the local signaling context.

The biological activities of IL-22 are transduced through several key intracellular signaling pathways. The specific pathway engaged, along with the cellular context, dictates the functional outcome of IL-22 signaling, a concept illustrated in **Figure 3**.

To avoid conflating cytokine production with receptor-proximal signaling, this section distinguishes pathways that regulate IL-22 production in immune cells from pathways activated after IL-22 engages the epithelial IL-22R1/IL-10R2 receptor complex. JAK1/TYK2-STAT3 represents the best-established receptor-proximal pathway. Evidence for NF-κB, mTOR, and MAPK involvement varies according to cell type and experimental model and is therefore described as direct downstream signaling, upstream regulation, or context-dependent crosstalk as appropriate.

### IL-22 signals through the JAK/STAT3 axis

The JAK/STAT pathway transduces cytokine-receptor engagement into transcriptional responses through receptor-associated janus kinases and cytoplasmic STAT proteins (119, 120). In the case of IL-22, binding to the IL-22R1/IL-10R2 receptor complex activates predominantly JAK1 and TYK2, followed by STAT3 phosphorylation, dimerization, and nuclear translocation (121). This receptor-proximal pathway should be distinguished from JAK/STAT activation by other cytokines in the inflamed intestine, including IL-6, IL-23, and interferons, which may modify IL-22 production or alter the epithelial response to IL-22 without being initiated by IL-22R itself (122). This cascade orchestrates transcriptional programs that govern epithelial barrier function, antimicrobial defense, and cell survival in intestinal epithelial cells.

#### Upstream regulatory network of IL-22-JAK/STAT signaling

IL-23 promotes IL-22 transcription in ILC3s by inducing the binding of a STAT3-STAT5 complex to the IL22 promoter, while STAT5 signaling contributes to the maintenance of intestinal epithelial integrity (123). Bacteroides fragilis strain ZY-312 has also been shown to promote mucosal regeneration in mice with DSS-induced colitis by increasing IL-22 secretion from ILC3s and enhancing STAT3 phosphorylation (24). Together, these findings suggest that microbiota-derived signals and the ILC3-STAT pathway coordinately regulate IL-22 production. The relative contribution and functional consequences of this regulatory network may vary according to disease phenotype, including UC and CD, as well as the local inflammatory microenvironment. After binding to its receptor complex, IL-22 signals predominantly through JAK1 and TYK2, which mediate the phosphorylation of downstream STAT proteins. JAK1 is broadly expressed across multiple cell types, whereas TYK2 has a prominent role in immune signaling and participates in pathways activated by IL-12, IL-23, and type I interferons (124). In the inflamed mucosa of patients with UC, phosphorylation of JAK2, JAK3, and TYK2, together with STAT family members including STAT1, STAT3, and STAT4, is significantly increased relative to non-inflamed mucosa, indicating broad activation of the JAK/STAT network (125). Although this study did not directly compare the expression or activation of JAK1 and TYK2 between UC and CD, IL-23 and other pro-inflammatory mediators are enriched in the intestinal mucosa of patients with CD, and IL-23 signaling is strongly dependent on TYK2. These observations suggest that TYK2 may carry greater functional weight in selected CD-associated inflammatory settings. Moreover, selective TYK2 inhibitors are currently being investigated for the treatment of IBD, further supporting the therapeutic relevance of this kinase in disease-associated signaling (124). Thus, the relative activation and functional contribution of JAK1 and TYK2 may differ between UC and CD according to tissue context and the prevailing cytokine environment. IL-22BP is a soluble, high-affinity antagonist of IL-22. Its expression is often inversely associated with IL-22 abundance, and it limits downstream JAK/STAT signaling by preventing IL-22 from binding to its membrane receptor complex (41). In patients with IBD, intestinal dendritic cells, eosinophils, and CD4+ T cells can all produce IL-22BP (43). Notably, IL-22BP levels are significantly higher in the inflamed ileum than in the colon of patients with CD, with mononuclear phagocytes and eosinophils representing the predominant cellular sources. In the inflamed colon, however, an elevated IL-22/IL-22BP ratio is strongly associated with the chemokine CCL24 (44). In UC, TNF-α induces IL-22BP expression, thereby restricting IL-22/STAT3-mediated mucosal repair and contributing to persistent inflammation (126). Conversely, anti-TNF treatment reduces IL-22BP levels, increases IL-22 bioavailability, and promotes mucosal healing. IL-22BP is also highly expressed by CD4+ T cells obtained from patients with IBD, whereas its expression in intestinal CD4+ T cells is significantly lower in patients who respond favorably to anti-TNF therapy (43). These findings suggest that IL-22BP differs between UC and CD in its tissue distribution and cellular origin. Its upregulation may limit the protective effects of IL-22 in the ileum of patients with CD, whereas in the UC colon, increased IL-22BP may contribute to disease persistence by restricting reparative STAT3 signaling.

Together, these findings indicate that activation of the IL-22-JAK/STAT pathway is determined by more than IL-22 production alone. Signals controlling IL-22 transcription, the cellular source of the cytokine, IL-22BP-dependent extracellular neutralization, receptor availability, and the broader JAK/STAT cytokine environment collectively determine the magnitude and duration of epithelial STAT activation. Once this regulatory threshold is crossed, the biological outcome depends on the downstream transcriptional program engaged in IL-22-responsive cells.

#### Functional divergence of downstream targets of IL-22-JAK/STAT signaling

The IL-22-JAK/STAT pathway dynamically regulates intestinal barrier function through a range of downstream effector genes in IBD. By activating STAT3, IL-22 induces the expression of antimicrobial peptides (AMPs) and thereby enhances mucosal host defense. In mouse models, the IL-22-STAT3 axis promotes the production of *Reg3β* and *Reg3γ*, which contribute to microbiota homeostasis and restrict bacterial translocation. For example, loss of the AIM2 inflammasome results in excessive IL-22 production, sustained STAT3 activation, and increased expression of *Reg3β* and *Reg3γ*. Although these changes promote crypt-cell proliferation, persistent activation may also increase the risk of colorectal tumorigenesis (127). In human IBD, the principal STAT3-dependent antimicrobial effectors include *REG3A* and α-defensins (DEFAs), whose expression patterns differ substantially from those observed in mice (128, 129). ATF3 represents an additional regulatory node in the IL-22-pSTAT3 signaling cascade. By negatively regulating protein tyrosine phosphatases such as SHP2 and PTP-Meg2, ATF3 enhances STAT3 phosphorylation and promotes AMP expression. Loss of ATF3 leads to Paneth-cell degeneration and depletion of AMP-containing granules, supporting an important role for ATF3 in the regulation of intestinal antimicrobial responses (110). The pronounced *Reg3* response observed in mouse models may therefore overestimate the extent of IL-22-mediated antimicrobial protection in humans, representing an important limitation in the clinical translation of findings from experimental models.

Claudin-2 is an important downstream target of IL-22-STAT3 signaling, and its function is highly context-dependent. Claudin-2 expression is significantly increased in the colon of patients with UC. As a pore-forming tight-junction protein, claudin-2 forms cation-selective channels that increase paracellular ion and water flux and may facilitate pathogen clearance, producing a “leaky but secretion-promoting” phenotype. Studies have shown that claudin-2-deficient mice develop more severe infectious or chemically induced colitis, whereas claudin-2 overexpression attenuates disease severity. These findings suggest that IL-22-induced claudin-2 may contribute to an adaptive epithelial response during acute injury (130). By contrast, claudin-2 expression is generally reduced in CD, particularly in ileal lesions, potentially because of differences in the local cytokine milieu and epithelial-cell composition. IL-13 can upregulate claudin-2 through IL-13 receptor α1, but this pathway may be attenuated in the ileum of patients with CD (131). In addition, inhibition of casein kinase 2 (CK2) blocks claudin-2 channel activity and ameliorates colitis in a claudin-2-dependent manner, suggesting that the therapeutic consequences of targeting claudin-2 must be evaluated according to disease subtype and tissue context (132). Certain interventions, including cedrol, a curcumin derivative, have also been reported to downregulate claudin-2 and restore barrier function in DSS-induced colitis models, further illustrating the context-dependent nature of this protein (133).

As a negative-feedback regulator of the JAK/STAT pathway, suppressor of cytokine signaling 3 (SOCS3) contributes to the control of IL-22 signaling. Myeloid-specific deletion of SOCS3 significantly aggravates DSS-induced colitis and is accompanied by increased monocyte and neutrophil infiltration in the colon and spleen, together with enhanced expression of colitis-associated genes in neutrophils. Notably, neutrophil depletion substantially ameliorates the disease phenotype in SOCS3-deficient mice, suggesting that the consequences of sustained STAT3 activation following SOCS3 loss are amplified by myeloid-cell-mediated inflammation (134). Concurrent activation of JAK2 and TYK2 and increased STAT3 phosphorylation in the inflamed mucosa of patients with UC suggest that negative-feedback mechanisms such as SOCS3-mediated inhibition may be insufficient to terminate STAT3 signaling effectively (125). IL-22 promotes intestinal epithelial regeneration and barrier repair through STAT3 activation; however, the absence of timely SOCS3-mediated inhibition may prolong STAT3 signaling excessively, leading to aberrant epithelial proliferation or the establishment of a tumor-promoting microenvironment (48). Dysregulated SOCS3 expression or function may therefore contribute to sustained STAT3 activity in UC, although whether this regulatory mechanism is similarly impaired in CD remains to be established.

In CD, sustained STAT1 activation promotes the expression of interferon-stimulated genes (ISGs), including CXCL9 and CXCL10, and contributes to a characteristic Th1-skewed inflammatory profile (135). Studies of patients with gain-of-function STAT1 mutations or loss-of-function STAT3 mutations have shown that peripheral blood mononuclear cells produce excessive CXCL10 in response to cytokine stimulation; this response can be partially reversed by the JAK inhibitor ruxolitinib. In addition, STAT1 promotes the expansion and persistence of pathogenic intestinal CD4+ T cells by upregulating Nlrc5, a regulator of MHC class I expression, thereby protecting these cells from natural killer cell-mediated clearance (136). In a model of crohn’s-like colitis, SOCS1 haploinsufficiency results in excessive JAK/STAT activation and the development of ileocolitis characterized by prominent STAT1-dependent inflammation. Treatment with a JAK1 inhibitor effectively ameliorates intestinal inflammation and reduces CD8+ T-cell infiltration (137). Collectively, these findings indicate that STAT1 not only transduces inflammatory signals in CD but also functions as a transcriptional regulatory node that sustains chronic immune activation.

STAT3 activation appears more closely associated with epithelial repair and antimicrobial defense in UC than in CD. IL-22 induces STAT3 phosphorylation in intestinal epithelial cells through the IL-22 receptor complex and subsequently promotes ATF3 expression. By inhibiting protein tyrosine phosphatases such as SHP2 and PTP-Meg2, ATF3 reinforces pSTAT3 signaling and ultimately promotes the production of antimicrobial peptides such as *REG3A (*110). Post-translational modification further regulates STAT3 activity. STAT3 palmitoylation, catalyzed by DHHC7, facilitates its recruitment to the plasma membrane and subsequent phosphorylation, whereas the depalmitoylating enzyme APT2 regulates its nuclear translocation. This dynamic modification cycle contributes to Th17-cell differentiation and the resolution of experimental colitis (138). STAT3 phosphorylation is also significantly increased in the inflamed mucosa of patients with UC. USP25 stabilizes pSTAT3 through deubiquitination and thereby contributes to the maintenance of epithelial barrier integrity, whereas reduced USP25 expression aggravates colitis (125, 139). STAT3 also exerts protective effects by regulating mitochondrial function and suppressing the expression of inducible nitric oxide synthase and fibrosis-associated mediators (140). Together, these mechanisms suggest that STAT3 activity in UC is preferentially associated with epithelial repair, antimicrobial defense, and the maintenance of mucosal homeostasis.

The precision use of JAK inhibitors and IL-22-targeted therapies will require a patient-stratification system based on molecular phenotypes. Available evidence indicates that pSTAT3 and pSTAT1 are co-activated in the inflamed mucosa of patients with UC, although STAT3 appears to be relatively dominant. By contrast, patients with CD exhibit stronger pSTAT1 activity and a more pronounced interferon-stimulated gene signature (136, 141). The pSTAT1/pSTAT3 ratio in intestinal biopsy specimens may therefore serve as a candidate biomarker for predicting responses to JAK inhibitors. For example, a greater reduction in STAT1 expression following tofacitinib treatment has been associated with a higher likelihood of clinical response (142). IL-22BP represents another potential biomarker because it is a major negative regulator of IL-22 activity. Its abundance in intestinal tissue or serum may provide information about the local intensity and functional state of IL-22 signaling. Patients with UC characterized by low IL-22BP expression and high pSTAT3 activity may potentially benefit from exogenous IL-22 or STAT3-biased agonists. Conversely, patients with CD characterized by low IL-22BP expression and high pSTAT1 activity may be more suitable candidates for selective JAK1/TYK2 inhibition or IL-22-neutralizing strategies. These proposed treatment groups remain to be validated prospectively. The kinase selectivity of different JAK inhibitors must also be considered, because differential targeting of JAK1, JAK3, or TYK2 may alter signaling through IL-10, IFN-β, and other cytokine pathways to varying degrees. Moreover, NOD2 polymorphisms can modify the sensitivity of immune cells to cytokine-induced STAT phosphorylation, suggesting that individual genetic background should also be incorporated into molecular stratification strategies (143).

### IL-22 signals through the NF-κB axis

NF-κB is activated by microbial products, inflammatory cytokines, and cellular stress and regulates immune-cell activation, epithelial survival, and inflammatory gene expression in the intestine. Its function is context-dependent: persistent activation can sustain mucosal inflammation, whereas complete disruption of epithelial NF-κB signaling can compromise tissue integrity. In the IL-22 network, the most clearly supported roles of NF-κB are upstream regulation of IL-22 production and parallel crosstalk with IL-22-activated STAT3; direct activation of NF-κB by epithelial IL-22R has not been established as a universal mechanism in IBD (144, 145).

Several mechanisms linking IL-22 activity to NF-κB signaling as follows:

#### Upstream regulation and feedback loops

ILC3s contribute to mucosal immune defense by producing IL-22 and IL-17. They also participate in lymphoid organogenesis, promote tissue protection and regeneration, and directly influence adaptive immune responses (146). Under physiological conditions, ILC3s help maintain host-microbiota symbiosis and immune tolerance by expressing major histocompatibility complex class II (MHC II) molecules and regulating intestinal epithelial responses (147). NF-κB signaling is closely involved in ILC3 activation, survival, and effector function. In the intestinal mucosa, neuropilin-1 (NRP1) promotes IL-17A production by ILC3s through a cell-intrinsic, NF-κB-dependent mechanism. Loss of NRP1 reduces ILC3 abundance and ameliorates colitis, supporting the involvement of NF-κB in the regulation of ILC3 function (148). Metabolites within the intestinal microenvironment can also shape ILC3 responses through NF-κB-related signaling. Acetate, a microbial product of dietary fiber fermentation, activates free fatty acid receptor 2 (FFAR2) on epithelial cells and indirectly modulates NF-κB activity in ILC3s, thereby promoting IL-22 secretion while reducing MHC II expression in colonic epithelial cells (149). Similarly, the Toll-like receptor 5 (TLR5) agonist CBLB502 induces IL-22 secretion in peripheral tissues through activation of the TLR5-NF-κB pathway, contributing to tissue protection (150). The effects of NF-κB activation are nevertheless context-dependent. A high-fat diet can rapidly disrupt intestinal ILC3 homeostasis, accompanied by aberrant NF-κB activation and dysregulated IL-22 secretion (151). NF-κB signaling also participates in the balance between ILC3 survival and cell death. TNF- or TL1A-dependent activation of caspase-8 can induce ILC3 death through the Fas-FasL pathway, and this process is closely associated with the regulation of NF-κB activity and IL-22 secretion. These observations illustrate the dual role of NF-κB in sustaining ILC3 viability and effector function while limiting excessive inflammatory activity (152).

#### Crosstalk between STAT3 and NF-κB

STAT3 and NF-κB interact at multiple levels within the intestinal inflammatory microenvironment. Under certain conditions, STAT3 activation can restrain the nuclear translocation and transcriptional activity of NF-κB, thereby contributing to anti-inflammatory responses and mucosal protection (153). In addition to canonical signaling mediated by phosphorylated STAT3, unphosphorylated STAT3 (uSTAT3) can interact with unphosphorylated NF-κB (uNF-κB) to form a noncanonical transcriptional complex that regulates downstream inflammatory genes (154). Interactions between NF-κB and other STAT-family proteins also affect epithelial barrier function. In intestinal epithelial cells, STAT5 suppresses myosin light-chain kinase (MLCK) expression through functional interaction with NF-κB, thereby helping to preserve epithelial integrity. Loss of STAT5 enhances NF-κB activation, reduces tight-junction protein expression, and ultimately impairs barrier function (155). These observations indicate that the effects of NF-κB cannot be interpreted independently of the broader STAT signaling environment. Several natural products and small-molecule compounds exert anti-inflammatory effects by acting on both pathways. For example, baicalin inhibits JAK2/STAT3 phosphorylation in T cells while suppressing NF-κB activation in macrophages, thereby alleviating intestinal inflammation in experimental colitis (156). Gramine has likewise been reported to inhibit the nuclear translocation of NF-κB and STAT3, resulting in anti-inflammatory and antiproliferative effects (153). Conversely, sustained activation of NF-κB in intestinal epithelial cells induces IBD-like pathological changes, including increased apoptosis and crypt hyperplasia. These findings suggest that epithelial NF-κB may represent a therapeutic target in settings characterized by persistent inflammation and excessive epithelial proliferation (153).

Within the nucleus, pSTAT3 and the NF-κB p65 subunit can interact either competitively or cooperatively, depending on the chromatin landscape and inflammatory context. These interactions reprogram gene transcription and may therefore influence the trajectory of inflammation and tissue repair (157, 158). In selected inflammatory settings, NF-κB binds specific enhancer regions and promotes histone H3 lysine 27 acetylation, thereby increasing chromatin accessibility and facilitating STAT3 recruitment to a subset of regulatory sites. This epigenetic cooperation enables the two pathways to coordinate the expression of shared target genes (158). pSTAT3 and RelA/p65 can also co-occupy the promoters of specific inflammatory genes, including IL17A, thereby enhancing transcription and establishing a positive-feedback circuit that sustains chronic inflammation (159). The lysine methyltransferase SMYD2 provides an additional layer of regulation by promoting the activation of both STAT3 and the NF-κB p65 subunit through methylation, thus linking epigenetic modification to inflammatory signaling (157). By contrast, negative regulators such as protein inhibitor of activated STAT3 (PIAS3) can suppress the activity of both STAT3 and NF-κB, forming a regulatory feedback mechanism that influences cell proliferation and tumor progression (160).

In UC, chronic intestinal inflammation is strongly associated with dysregulated NF-κB signaling. Non-coding RNAs can influence disease progression by modulating the expression of multiple components of this pathway (161). Persistent NF-κB activation compromises intestinal epithelial integrity, increases paracellular permeability, and contributes to the maintenance of chronic colonic inflammation (162). These findings provide a mechanistic context in which NF-κB may modify epithelial responses to IL-22-activated STAT3, although simultaneous activation of the two pathways does not necessarily establish direct activation of NF-κB downstream of IL-22R.

A distinct signaling context has been reported in active colonic CD. In colonic epithelial cells from these patients, IL-22 induces an ER stress-response transcriptional program, and both the IL-22-response module and the ER stress module are enriched in affected tissues. Blockade of IL-22 or the IL-12/IL-23 pathway alleviates epithelial ER stress, supporting a potentially pathogenic role for IL-22 under defined inflammatory conditions (23). This observation does not imply that IL-22 is uniformly detrimental in CD; rather, it illustrates how the epithelial stress state and concurrent inflammatory pathways can alter the outcome of IL-22 signaling.

More broadly, dysregulated NF-κB signaling can connect microbial imbalance with intestinal inflammation and epithelial barrier dysfunction (163). In settings of persistent pathway activation, direct modulation of epithelial NF-κB may help reduce apoptosis, inflammation, and crypt hyperplasia, offering a potential therapeutic strategy for refractory IBD (164). The noncanonical pathway may also contribute to disease. NF-κB-inducing kinase (NIK), a central component of noncanonical NF-κB signaling, has been implicated in IBD pathogenesis, and systemic administration of small-molecule NIK inhibitors reduces inflammation and improves colonic histopathology in several experimental colitis models (165). Additional regulatory mechanisms operate at the transcriptional, epigenetic, and post-transcriptional levels. The long non-coding RNA ANRIL competitively binds Yin Yang 1, thereby restricting its interaction with the NF-κB p65 subunit and establishing an ANRIL/p65 negative-feedback loop. This mechanism may provide a nucleic acid-based strategy for preserving intestinal barrier function (166). The RNA methyltransferase METTL3 also contributes to inflammatory signaling by promoting p65 phosphorylation; accordingly, METTL3 knockdown or pharmacological inhibition of NF-κB markedly reduces inflammation (167). Thus, therapeutic modulation of NF-κB is no longer limited to conventional anti-inflammatory agents. Small-molecule inhibitors and non-coding RNA-based approaches are being explored as more targeted means of regulating this pathway (161).

### IL-22 signals through the mTOR axis

mTOR integrates nutrient availability, growth-factor signaling, cellular energy status, and immune activation through the mTORC1 and mTORC2 complexes (168, 169). In the IL-22 network, mTOR may operate at two different levels: as an upstream regulator of cytokine production in immune cells and as a possible downstream effector in IL-22-responsive cells. Evidence for the first role is available in mucosal immune populations, whereas direct evidence for an IL-22R-PI3K-AKT-mTOR pathway in intestinal epithelial cells remains more limited.

#### Upstream regulation of IL-22 production

The PI3K-AKT-mTOR pathway influences IL-22 production by regulating the differentiation, metabolic fitness, and translational capacity of IL-22-producing lymphocytes. Roquin has been implicated in the control of Treg-cell function and in restraining aberrant differentiation of T follicular helper (Tfh) and Th17 cells through the PI3K-AKT-mTOR pathway. Roquin deficiency enhances AKT-mTOR activity and promotes Th17-cell differentiation, whereas pharmacological inhibition of PI3K or mTOR reverses this phenotype, supporting a role for this pathway in the regulation of Th17-cell development and function (170).

Within Th17 cells, glutathione availability promotes IL-22 protein synthesis by maintaining mitochondrial function and intracellular signaling. Deletion of Gclc reduces glutathione availability, suppresses PI3K-AKT-mTOR activity, and decreases 4E-BP1 phosphorylation, thereby limiting IL-22 translation and compromising intestinal barrier integrity (171). TGF-β has also been reported to promote IL-22 production by Th17 cells through AhR induction and activation of PI3K signaling (172). Together, these findings indicate that IL-22 output is regulated not only at the transcriptional level but also through metabolically sensitive translational mechanisms.

mTOR signaling also shapes ILC3 homeostasis and effector function. Both mTORC1 and mTORC2 have been implicated in the regulation of ILC3 maintenance and pathological immune responses during colitis. Disruption of mTOR signaling reduces ILC3 proliferation, attenuates activation-induced IFN-γ production, and decreases the release of inflammatory mediators (173).

#### Downstream signaling and context-dependent outcomes

In the intestinal epithelium, mTOR signaling—particularly mTORC1 activity—is enriched in stem and progenitor cells located near the crypt base, where it regulates cell proliferation, metabolic reprogramming, and lineage differentiation (174–176). Following intestinal injury, controlled and self-limited mTORC1 activation supports crypt stem-cell division, epithelial dedifferentiation, and regenerative responses (175, 176). mTOR signaling may also cooperate with IL-22-associated programs to maintain the intestinal barrier by regulating epithelial differentiation and tight-junction protein expression (177, 178). The available evidence therefore supports functional convergence between IL-22 and mTOR during epithelial repair. However, the extent to which mTOR is activated directly downstream of IL-22R1/IL-10R2, rather than indirectly through STAT3-dependent growth-factor or metabolic circuits, may vary according to the experimental system. Within these boundaries, coordinated IL-22 and mTOR activity may help regulate the balance between proliferation and differentiation in intestinal stem and progenitor cells during post-injury repair (175, 179).

Pharmacological modulation of mTOR has shown beneficial effects in several animal models. Rapamycin-mediated inhibition of mTOR alleviates colitis, reduces ILC3 proliferation, and limits the production of inflammatory mediators (173). In DSS-induced experimental colitis, rhein suppresses PI3K-AKT-mTOR pathway activity, as reflected by reduced phosphorylation of PI3K, AKT, mTOR, and p70S6K1, together with improvements in colon length, body-weight loss, and pro-inflammatory cytokine production (180). In the same general model, *Gypensapogenin* I, a saponin component derived from *Gynostemma pentaphyllum*, has been reported to regulate PI3K-AKT signaling through AKT1, attenuate disease progression, and restore epithelial barrier function (181). Dynamic analyses further indicate that mTOR inhibition can modify epithelial differentiation and, in turn, reshape the colonic cytokine environment. These findings suggest that epithelial differentiation is closely linked to colonic homeostasis and inflammatory cytokine production (177). These animal studies provide evidence that mTOR-directed interventions can modify intestinal inflammation, epithelial differentiation, and immune-cell function. They do not, however, establish that all therapeutic effects are mediated through IL-22 or through signaling directly downstream of IL-22R.

Human IBD data provide a separate level of evidence. In patients with UC, colonic epithelial mTORC1 activity is positively correlated with cyclooxygenase-2 (COX-2) expression, linking abnormal mTORC1 activation to an inflammatory epithelial phenotype (182). Analyses of clinical tissue samples have also identified increased expression of mTOR pathway components, in the colonic mucosa of patients with UC, suggesting that mTORC2-related signaling may also be dysregulated during disease progression (183). In colonic tissues from patients with active CD, both an IL-22-responsive transcriptional module and an ER stress-response module are enriched. The IL-23/IL-17 pathway also contributes to the inflammatory environment, with IL-23 promoting the expansion of pathogenic Th17 cells and inducing several inflammatory mediators, including IL-22 (23). Differences between UC and CD are also evident in the phenotype of Th17 cells. Th17 populations isolated from the mesenteric lymph nodes of patients with CD and UC differ in both frequency and transcriptional characteristics. Th17 cells from patients with CD display greater expression of pathogenic gene signatures, whereas those from patients with UC show relatively higher expression of genes associated with non-pathogenic states (184). These observations suggest that Th17-cell plasticity and its upstream regulatory networks may diverge between the two diseases before these cells are recruited to the inflamed colon. Nevertheless, direct human studies simultaneously measuring mTOR activity, IL-22 production, IL-22BP abundance, and epithelial IL-22R signaling remain limited.

### IL-22 signals through the MAPK axis

The MAPK network comprises ERK, p38, and c-Jun N-terminal kinases (JNK) signaling modules that regulate cell proliferation, stress responses, cytokine production, and epithelial adaptation (185). In IBD, hyperactivation of JNK and p38 pathways substantially amplifies pathogenesis through heightened production of pro-inflammatory cytokines and extensive crosstalk with NF-κB signaling (186). Oxidative stress, a hallmark of chronic intestinal inflammation, potently drives this aberrant activation (187).

However, the interplay between IL-22 and MAPK cascades is bidirectional and contextually nuanced.

#### Upstream MAPK regulation

Within the intestinal immune microenvironment, ILC3 activation and IL-22 production are regulated by multiple cytokines and intracellular signaling pathways. MAPK signaling contributes to ILC3 effector function and the production of IL-22. For example, *Bacillus anthracis* lethal toxin disrupts ILC3 activity by interfering with MAPK signaling, reducing ERK1/2 and p38 phosphorylation and thereby suppressing IL-23-induced IL-22 production (188). This finding places ERK1/2 and p38 upstream of IL-22 secretion in ILC3s and distinguishes their function in cytokine-producing immune cells from MAPK activity in epithelial cells after IL-22 receptor engagement.

Th22 cells and IL-22-producing Th17 cells share elements of an IL-6-dependent developmental program, while IL-17 signaling has also been reported to promote Th22-cell development (189). MAPK signaling contributes to the differentiation and effector functions of these T-cell populations. For example, allicin reduces the production of inflammatory mediators, including IL-22, by suppressing IL-17-induced TRAF6-MAPK-NF-κB and STAT3-NF-κB signaling (190). Punicalagin has similarly been reported to decrease IL-22 and other inflammatory mediators through inhibition of MAPK/ERK and NF-κB signaling (191).

MAPK signaling intersects with IL-22 biology at both upstream and downstream levels. In immune cells, inhibition of MAP2Ks, followed by reduced ERK1/2 and p38 activation, markedly impairs IL-23-induced IL-22 production by ILC3s (188). Th22 cells, which produce IL-22 and participate in mucosal defense and tissue repair, have also been associated with MAPK activation (192).

#### Downstream MAPK activation

Evidence from intestinal epithelial models indicates that IL-22 can influence barrier function through MUC13-associated ROCK2/MAPK signaling (26). MAPK activity also contributes more broadly to epithelial survival and barrier integrity. For example, inhibition of p38 MAPK in intestinal epithelial cells markedly increases TNF-α-induced apoptosis and necroptosis, resulting in intestinal injury (193). These experiments show that pathogenic toxin-mediated inhibition of MAPK, but not STAT3, impairs IL-23-induced IL-22 production in ILC3s. By contrast, in epithelial cells that have already encountered IL-22, interactions between STAT3 and MAPK-related signaling may shape the response to local inflammatory and metabolic conditions (188).

During acute mucosal injury or infection, IL-22 generally supports epithelial protection and repair. These effects may be accompanied by modulation of excessive MAPK activity and other inflammatory pathways. Administration of IL-22 has been reported to alleviate experimental colitis, improve epithelial barrier function, and promote restoration of intestinal homeostasis (194). In a model of radiation-induced enteropathy, inhibition of epithelial p38 MAPK regulated metallothionein expression and strengthened intercellular junctions, thereby improving barrier integrity and mucosal healing (195). Certain natural compounds have likewise reduced epithelial inflammatory mediator expression and abnormal mucus secretion by suppressing NF-κB and MAPK activation (196). MAPKs also regulate intestinal inflammation independently of IL-22. In experimental IBD models, inhibition of TAB1/MAP2K4-centered signaling or blockade of p38 phosphorylation restores the Th17/Treg balance and reduces intestinal injury (197, 198). In CD4+ T-cell-mediated intestinal inflammation, MAP3K2 influences T-cell differentiation and colitis severity through the JNK pathway (199). These latter observations define the wider inflammatory environment in which IL-22 acts but do not constitute direct evidence of epithelial IL-22R-MAPK signaling.

Evidence relevant to UC comes from both experimental colitis models and human observations, and these sources should be interpreted separately. In experimental models relevant to colonic inflammation, GPR35 activation promotes fibronectin and integrin expression in colonic epithelial cells through ERK1/2, thereby supporting mucosal repair (200). Certain traditional Chinese medicine extracts have also been reported to improve barrier function and reduce inflammation through EGFR-dependent MAPK/ERK1/2 signaling in colitis models (201). Rutin has also been shown to alleviate chronic experimental colitis and improve colonic epithelial integrity through modulation of the p38/MK2 pathway (202). In human UC, MAPK15 expression has been reported to be reduced in affected tissue. Experimental overexpression of MAPK15 attenuates intestinal inflammation and cellular senescence by limiting mitochondrial-fission-associated injury through the SUZ12/DRP1 axis (203). Collectively, these findings implicate several MAPK modules in UC-associated epithelial injury and repair. However, the available evidence does not yet show that ERK1/2, MAPK15, and p38/MK2 are uniformly activated downstream of IL-22R in human UC. They are more appropriately interpreted as epithelial pathways that may intersect with IL-22 responses in a disease- and model-dependent manner.

CD presents a different pathological setting, characterized not only by transmural inflammation but also, in selected patients, by granuloma formation, intestinal fibrosis, strictures, and penetrating complications. Within this setting, IL-22 and MAPK-related signaling may contribute to epithelial stress, immune activation, and tissue remodeling through distinct but potentially convergent mechanisms. Intestinal fibrosis develops after prolonged inflammatory stimulation drives the production of growth factors and profibrotic cytokines, activates immune and non-immune populations such as macrophages and myofibroblasts, and promotes excessive extracellular-matrix deposition and stricture formation (204, 205). Transcriptomic analyses have identified MAPK-related regulatory networks in CD, and their activation may be associated with interactions between disease-associated microbial communities and host-cell receptors (206). These human findings support the involvement of MAPK-associated inflammatory networks in complicated CD, but they do not establish a linear IL-22R-MAPK-fibrosis pathway. Direct studies simultaneously measuring IL-22, IL-22BP, epithelial IL-22R activity, specific MAPK modules, and fibrosis-related outputs will be required to define the degree of mechanistic coupling. Animal models have provided much of the mechanistic evidence concerning IL-22 and MAPK signaling, but their findings cannot be transferred directly to human IBD.

Differences between experimental and human observations illustrate this limitation. For example, affected mucosa from patients with collagenous colitis reportedly showed neither increased IL-22 nor altered MAPK signaling, in contrast to the marked pathway activation described in several animal models of colitis (207). Collagenous colitis is distinct from UC and CD, so these findings should not be treated as direct evidence against MAPK involvement in IBD; instead, they emphasize that IL-22 and MAPK responses vary across inflammatory intestinal disorders. Moreover, the tissue microenvironment and microbiome composition of human IBD cannot be reproduced fully in conventional animal models. Human multi-omics studies provide an additional level of evidence. Longitudinal analyses have identified functional and molecular perturbations of the intestinal microbiome during active IBD. Mucosa-associated microbial communities from newly diagnosed patients may also show spatial patterns and disease-discriminating profiles that differ from those obtained from stool samples (4, 208). Future studies should therefore integrate human single-cell transcriptomics, intestinal organoids, spatially resolved analyses, and gut-on-a-chip or other advanced mucosal modeling platforms to bridge the gap between experimental models and clinical disease.

The multifaceted downstream signaling pathways of IL-22 are summarized in **Table 2**.

**Table 2:** Signaling pathways that regulate or interact with IL-22 biology in IBD.

Signal pathway	Position relative to IL-22	Principal target cell(s)	Experimental model	Disease context	Human IBD evidence	Direction of effect	Evidence strength and main limitation	References
JAK1/TYK2-STAT3	Canonical direct signaling downstream of epithelial IL-22R1/IL-10R2	IECs, crypt stem/progenitor cells, and other IL-22-responsive non-hematopoietic cells	Experimental colitis, epithelial systems, organoids, and immune-cell studies	Acute repair, epithelial defense, chronic inflammation, UC and CD	UC: increased JAK/STAT activity and IL-22-associated CXCR2-ligand expression; CD: IL-22-associated epithelial ER-stress program and STAT1-rich inflammatory context	IL-22 activates JAK1/TYK2-STAT3, promoting antimicrobial programs, epithelial survival, and repair; sustained activity can promote chemokines, neutrophil recruitment, ER stress, or abnormal proliferation	Strongest direct IL-22R downstream pathway; biological outcome depends on IL-22BP, signaling duration, target-cell state, and concurrent inflammatory signals	(19, 23, 110, 121, 125–127, 135–143)
NF-κB	Upstream regulator of IL-22 production; parallel epithelial crosstalk with STAT3	ILC3s, Th17-associated immune populations, IECs, macrophages	ILC3 studies, experimental colitis, pharmacological studies, and non-intestinal transcriptional models	Intestinal immune activation, chronic colitis, UC and CD	UC: persistent epithelial NF-κB activation and non-coding-RNA-associated dysregulation; CD: limited direct paired IL-22/NF-κB human evidence	NF-κB can promote IL-22 production through inflammatory cytokines and direct IL22-promoter regulation; in IECs, NF-κB may cooperate with STAT3 during chronic inflammation	Moderate for upstream immune-cell regulation; indirect for epithelial IL-22R-associated crosstalk. NF-κB should not be presented as a universal direct IL-22R output	(144, 145, 154–156, 158–160)
PI3K-AKT-mTOR	Upstream regulator of IL-22-producing immune cells; potential downstream or convergent pathway in epithelial cells	Th17 cells, ILC3s, IECs, crypt stem/progenitor cells	Th17 and ILC3 studies; DSS colitis; intestinal regeneration models	Experimental colitis, epithelial repair, UC and CD	UC: epithelial mTORC1 activity correlates with COX-2; CD: pathogenic Th17 signatures, IL-23/IL-17 activity, and IL-22-associated epithelial stress	mTOR supports immune-cell metabolic fitness and IL-22 translation; epithelial mTOR may converge with IL-22-associated repair programs, whereas sustained activity may promote pathological inflammation or proliferation	Moderate for upstream IL-22 regulation; limited/model-dependent evidence for direct IL-22R-mTOR signaling in IECs	(170–184)
ERK/p38/JNK MAPK	Upstream regulator of IL-22 production; selected downstream or convergent epithelial signaling	ILC3s, Th17/Th22 cells, IECs	ILC3 toxin-perturbation model, experimental colitis, IEC models, and non-intestinal epithelial studies	Experimental colitis, epithelial injury/repair, UC and complicated CD	UC: MAPK-related epithelial injury/repair pathways; CD: MAPK-related inflammatory and fibrotic networks, but limited direct IL-22R comparison	ERK1/2 and p38 support IL-23-induced IL-22 production in ILC3s; MUC13-associated ROCK2/MAPK signaling may modify IL-22-related epithelial barrier responses	Moderate for upstream ILC3 regulation; limited direct intestinal evidence downstream of IL-22R. Skin and other non-intestinal models are extrapolative	(26, 188, 190–193, 195–203, 205, 206, 208)

“Position relative to IL-22” distinguishes mechanisms that regulate IL-22 production in immune cells from signaling events occurring after IL-22 engages epithelial IL-22R1/IL-10R2. JAK1/TYK2-STAT3 is the best-established receptor-proximal pathway. NF-κB, mTOR, and MAPK pathways may regulate IL-22 production, operate in parallel with IL-22-activated STAT3, or modify epithelial responses in a cell- and model-dependent manner.

## Disease-specific differences between UC and CD

Although UC and CD share microbial dysbiosis, altered metabolite availability, epithelial barrier dysfunction, and dysregulated cytokine signaling, these abnormalities arise within distinct anatomical and inflammatory environments. UC is confined to the colon and is characterized predominantly by continuous mucosal inflammation, whereas CD can involve any segment of the gastrointestinal tract and frequently exhibits discontinuous and transmural disease. These differences influence microbial habitat, metabolite exposure, epithelial-cell composition, and the signaling environment in which IL-22 acts.

IL-22 also operates within different immunological and epithelial contexts in UC and CD. In UC, IL-22 can activate epithelial STAT3-dependent programs that support repair but may also induce C-X-C motif chemokine receptor 2 (CXCR2) ligands, including CXCL1, CXCL5, and CXCL8, thereby promoting neutrophil recruitment; increased activity of this program has been associated with resistance to ustekinumab therapy (19). In active colonic CD, by contrast, IL-22-responsive transcription has been linked to epithelial ER stress, while a stronger STAT1- and interferon-associated environment may modify the consequences of IL-22-driven STAT3 activation (23, 135–137). These observations do not indicate that IL-22 is uniformly pathogenic in either disease. Rather, they suggest that the same cytokine may activate different transcriptional outputs according to epithelial stress, concurrent cytokine signaling, disease activity, and anatomical location. The interpretation of IL-22 abundance is further complicated by IL-22BP. In CD, IL-22BP production is heterogeneously distributed between the inflamed ileum and colon and involves different cellular sources, whereas TNF-dependent IL-22BP induction may restrict IL-22-mediated repair in inflamed colonic tissue. Consequently, comparisons based on circulating or mucosal IL-22 alone may not accurately reflect effective IL-22R1/IL-10R2 signaling.

Disease-specific differences in the noncanonical pathways interacting with IL-22 are less clearly defined. NF-κB is dysregulated in both UC and CD, but few human studies have measured NF-κB activity together with IL-22, IL-22BP, and epithelial STAT signaling in matched samples. Human UC studies have identified abnormal epithelial mTORC1- and mTORC2-related activity, whereas findings in CD more often emphasize pathogenic Th17 states, IL-23-driven inflammation, and IL-22-associated epithelial stress (23, 182–184). MAPK-related pathways contribute to epithelial injury and repair in UC and may intersect with inflammatory and fibrotic networks in complicated CD; however, current evidence does not establish a disease-specific linear IL-22R-MAPK pathway or an IL-22R-MAPK-fibrosis axis (26, 203–206). These distinctions have therapeutic implications. Strategies intended to enhance IL-22-mediated repair may be more appropriate when epithelial regeneration is impaired and inflammatory chemokine programs are limited, whereas increasing IL-22 activity in tissues characterized by high IL-22 availability, STAT1/NF-κB activation, neutrophil recruitment, or ER stress could be ineffective or harmful. Patient stratification should therefore integrate disease type and location with metabolite profiles, IL-22/IL-22BP balance, receptor availability, and downstream pathway activity rather than relying on diagnosis or IL-22 concentration alone.

## Limitations

Several limitations should be considered when interpreting the microbiota-metabolite-IL-22 framework presented in this review. First, although a structured literature search and qualitative evidence-appraisal strategy were applied, this article remains a narrative review rather than a systematic review. No formal risk-of-bias assessment or quantitative evidence synthesis was performed, and selection bias cannot therefore be excluded. The available literature is also highly heterogeneous with respect to study design, analytical platform, biological sample, disease phenotype, and experimental model, which limits direct comparison across studies.

Current human studies of IBD have largely examined individual signaling pathways or the local expression of selected cytokines, with few studies directly and simultaneously comparing IL-22 with multiple downstream pathways, including JAK/STAT, NF-κB, mTOR, and MAPK. For example, a biopsy-based analysis of colonic mucosa from patients with UC assessed the phosphorylation of JAK1, JAK2, JAK3, TYK2, STAT1, STAT3, and STAT4 (125). These molecules were markedly upregulated in inflamed tissue and displayed coordinated activation and sex-related differences; however, neither IL-22 abundance nor the activation states of other relevant pathways were evaluated in parallel. Similarly, single-cell transcriptomic analysis identified enhanced JAK-STAT signaling and increased expression of IL-6, IL-12, and IL-23 in terminal ileal macrophages from patients with CD, but did not provide a comprehensive cross-pathway comparison (208). Other human studies have shown that CD4+ T cells from patients with IBD produce high levels of IL-22BP, whose expression can be modulated by anti-TNF-α treatment, and that mucosal kinase activity profiles differ between UC and CD and may predict responses to tofacitinib (43, 209). Nevertheless, these findings remain fragmented across separate cohorts and experimental settings. In the absence of direct studies that simultaneously quantify IL-22 concentrations and the phosphorylation states of multiple downstream pathways within the same patient cohort, the complete regulatory architecture of the microbial metabolite-IL-22-multipathway axis cannot yet be defined precisely in humans.

Animal models have contributed substantially to the understanding of interactions between the intestinal microbiota and host immunity, yet their translation to human pathophysiology remains limited and, in some respects, contested. In experimental models, IL-22 is commonly associated with mucosal protection and epithelial regeneration. For instance, selected bacterial strains can alleviate DSS-induced colitis by promoting IL-22 secretion from ILC3s and enhancing STAT3 phosphorylation (24). In the more heterogeneous inflammatory environment of human disease, however, IL-22 displays dual biological effects. Sustained IL-22 activity may contribute not only to persistent colitis but also to an increased risk of colitis-associated carcinogenesis, particularly in patients with long-standing IBD (48). Moreover, mechanisms established in animals may not apply directly to human disease. BTK deficiency, for example, accelerates colitis in mice independently of changes in the intestinal microbiota; instead, disease progression is driven by increased IL-12 secretion from dendritic cells and a shift toward Th1 differentiation among T cells (210). This mechanism differs from the prominent contribution of microbial dysbiosis to human IBD. Sustained epithelial NF-κB activation in animal models can also induce crypt hyperplasia and Paneth-cell depletion, accompanied by aberrant Wnt signaling and an increased risk of malignancy (164). In humans, however, the boundary between epithelial-cell-intrinsic inflammation and neoplastic transformation, together with the optimal window for therapeutic intervention, remains uncertain. These differences limit the direct application of animal findings to precision clinical management.

Gut microbial metabolite concentrations also vary substantially across intestinal regions and over time. Most available studies have examined the linear effect of a single metabolite on an individual signaling pathway, leaving the nonlinear regulation of interconnected pathway networks by fluctuating metabolite concentrations insufficiently characterized. Existing evidence indicates that diet-microbiota interactions can alter metabolite availability and thereby influence mTOR, MAPK, STAT3, and NF-κB signaling (211). For example, the microbial metabolite ellagic acid can activate the AhR, trigger the NLRP6 inflammasome, and subsequently induce IL-22 expression to strengthen intestinal barrier function (212). Conversely, depletion of endogenous glutathione in Th17 cells suppresses PI3K/AKT/mTOR signaling and limits IL-22 translation (171). Ginsenoside Rk3 can likewise restrain excessive JAK-STAT3 activation by modulating amino acid and bile acid metabolism (213). Most of these findings, however, were obtained using fixed metabolite concentrations *in vitro* or single-time-point interventions in animals. Such designs do not reproduce the dynamic fluctuations that occur within the intestinal lumen or reveal their nonlinear effects, activation thresholds, and feedback inhibition across the JAK/STAT, NF-κB, mTOR, and MAPK pathways. This unresolved mechanistic dimension makes it difficult to determine how physiological or pathological peaks and troughs in metabolite concentrations shape downstream IL-22 signaling and limits the development of precision interventions guided by dynamic metabolite monitoring.

## Conclusion and future perspectives

Intestinal microorganisms and their metabolic products contribute to the initiation, persistence, and phenotypic heterogeneity of IBD (214). SCFAs, Trp-derived metabolites, and secondary BAs can influence mucosal immunity and IL-22 biology through AhR, GPR, nuclear receptors, and metabolically responsive intracellular pathways (30, 215). IL-22 translates these microbial and immune signals into epithelial responses primarily through the IL-22R1/IL-10R2-JAK1/TYK2-STAT3 pathway, thereby regulating antimicrobial defense, epithelial survival, regeneration, and mucus-associated barrier functions (18, 48, 110). NF-κB, mTOR, and MAPK signaling also intersect with IL-22 biology, but their positions within this network differ: they may regulate IL-22 production in immune cells, operate in parallel with IL-22-activated STAT3, or modify epithelial responses in a model- and context-dependent manner (216–218). The functional consequences of this network differ between disease phenotypes and anatomical sites. In CD, for example, the heterogeneous distribution of IL-22BP between inflamed ileal and colonic tissues may alter the amount of IL-22 available for epithelial receptor engagement. Altered Trp metabolism, including changes in kynurenine-pathway metabolites and NAD+ homeostasis, may further modify epithelial stress and inflammatory responses (43, 94, 219). In UC, IL-22-dependent STAT3 activity may support epithelial repair but can also induce CXCR2 ligands and promote neutrophil recruitment in an established inflammatory environment. These observations indicate that neither IL-22 concentration nor the abundance of an individual metabolite is sufficient to predict biological outcome (19). The local IL-22/IL-22BP balance, epithelial target-cell state, anatomical location, concurrent inflammatory pathways, and disease activity must be considered together when interpreting this network.

Metabolite-directed interventions offer several preclinical opportunities. Dietary fiber and prebiotic supplementation can increase microbial SCFA production, whereas selected probiotic strains, postbiotic preparations, and dietary substrates may enhance the generation of indole-type AhR ligands and support IL-22-dependent mucosal defense (30, 212, 220). AhR agonists and defined metabolite formulations may provide greater pharmacological control than broad dietary interventions, but their long-term effects will require careful evaluation because sustained AhR activation can produce ligand-, cell-, and tissue-specific responses. Several barriers currently limit clinical testing of these interventions. Microbial metabolite exposure is shaped by baseline microbiome composition, diet, medication, intestinal transit, hepatic metabolism, renal clearance, and disease location. The dose measured in stool or plasma may not reflect the concentration reaching IL-22-producing immune cells or epithelial target cells. Orally delivered metabolites may also be absorbed or transformed before reaching the affected intestinal segment, while probiotics and dietary interventions may produce variable effects across individuals. Early clinical studies should therefore use phenotype-stratified cohorts, intestinally targeted delivery when feasible, and paired measurements of metabolite exposure, mucosal IL-22/IL-22BP, epithelial signaling, and endoscopic healing rather than relying on symptom scores alone.

JAK inhibition requires a similarly context-sensitive strategy (221, 222). Tofacitinib, which inhibits multiple JAK-family members, is approved for moderate-to-severe UC and offers several practical advantages over biologic agents, including oral administration, a relatively short half-life, rapid onset of action, and lack of anti-drug immunogenicity (222, 223). In UC, tofacitinib suppresses mucosal STAT1, STAT3, and STAT5 activity and reduces cytokine programs associated with Th1, Th2, and Th17 responses (224). Its clinical development in CD was discontinued after pivotal trials failed to meet their primary efficacy endpoints, illustrating that JAK-dependent inflammatory programs and drug responses cannot be assumed to be equivalent in UC and CD (225). Newer agents seek to improve the balance between efficacy and systemic safety through kinase selectivity or intestinal restriction (222). Upadacitinib preferentially targets JAK1 and has demonstrated efficacy in both UC and CD, whereas filgotinib has been approved for UC in selected jurisdictions and has also been evaluated in CD (223, 225). Dual JAK1/TYK2 inhibitors, such as PF-06700841, and more selective TYK2 inhibitors may be relevant to patients with prominent IL-12/IL-23- or interferon-associated signaling (226). Gut-restricted inhibitors such as TD-1473 were developed to achieve high intestinal exposure with limited systemic concentrations, and early clinical studies provided information on tolerability and local pathway inhibition (227). Ivarmacitinib, another selective JAK1 inhibitor, has also shown efficacy and acceptable short-term tolerability in clinical studies of moderately to severely active UC (228).

NF-κB and MAPK activity may decline indirectly when JAK inhibition reduces the inflammatory cytokine environment, but neither pathway is a direct selective target of JAK inhibitors. Combination treatment with an NF-κB-, mTOR-, or MAPK-directed agent may be considered only when a corresponding pathway is demonstrably active and when additive toxicity is carefully monitored. Potential combinations—including anti-TNF plus anti-IL-23 treatment or a metabolite-directed intervention plus carefully titrated JAK inhibition—should initially be evaluated in biomarker-defined experimental systems and early-phase clinical studies (12, 229).

No single biomarker is likely to capture the state of this network. For example, high IL-22 accompanied by high IL-22BP may indicate limited effective cytokine availability, whereas high IL-22 with low IL-22BP, strong pSTAT1 or NF-κB activity, and elevated neutrophil-recruiting chemokines may identify a subgroup in which IL-22 participates in inflammatory amplification. Conversely, low concentrations of AhR ligands, reduced epithelial pSTAT3, and impaired regenerative markers may indicate deficient mucosal-repair signaling. A composite profile integrating metabolite availability, IL-22 bioavailability, receptor expression, pathway activation, and disease location is therefore more biologically informative than any isolated measurement.

Multi-omics technologies provide a practical framework for constructing such profiles. Integration of genomics, bulk and single-cell transcriptomics, proteomics, epigenomics, and metabolomics can resolve disease-associated cell states and identify microbial and host features linked to activity, complications, and treatment response (230). Fecal multi-omics approaches combining 16S rRNA sequencing, metagenomics, metatranscriptomics, metabolomics, and metaproteomics can characterize microbial functions that are not apparent from taxonomic composition alone. These data should be integrated with mucosal measurements because stool and tissue-associated microbial communities may provide different information (231). Longitudinal sampling is also necessary to distinguish biomarkers that precede disease activity or therapeutic response from those that merely reflect established inflammation. The systematic integration of multi-omics data with clinical phenotypes and environmental exposures may identify novel biomarkers of response to biologic therapies and small-molecule drugs, while also revealing underlying molecular patterns that inform precision medicine and personalized treatment strategies (232, 233).

## Glossary

IBD: Inflammatory bowel disease
CD: Crohn’s disease
UC: Ulcerative colitis
SCFAs: Short-chain fatty acids
BAs: Bile acids
Trp: Tryptophan
TMAO: Trimethylamine N-oxide
TMA: Trimethylamine
IL: Interleukin
ILCs: Innate lymphoid cells
GPR: G protein-coupled receptor
TGR5: Takeda G protein-coupled receptor 5
AhR: Aryl hydrocarbon receptor
JAK/STAT: Janus kinase/signal transducer and activator of transcription
ROS: Reactive oxygen species
ILC3: Group 3 innate lymphoid cell
Th: T helper cell
NF-κB: Nuclear factor kappa B
mTOR: Mechanistic target of rapamycin
MAPK: Mitogen-activated protein kinase
ER: Endoplasmic reticulum
HDAC: Histone deacetylase
ERK: Extracellular signal-regulated kinase
HIF1α: Hypoxia-inducible factor 1α
CYP7A1: Cholesterol 7α-hydroxylase
CA: Cholic acid
CDCA: Chenodeoxycholic acid
DCA: Deoxycholic acid
LCA: Lithocholic acid
TCA: Taurocholic acid
GCA: Glycocholic acid
UDCA: ursodeoxycholic acid
TUDCA: Tauroursodeoxycholic acid
THDCA: Taurohyodeoxycholic acid
ISCs: Intestinal stem cells
KP: Kynurenine pathway
5-HT: 5-hydroxytryptamine
IDO1/IDO2: indoleamine 2, 3-dioxygenase
IPA: Indole-3-propionic acid
IAA: Indole-3-acetic acid
IAld: Indole-3-aldehyde
TDO: Tryptophan 2, 3-dioxygenase
TPH: Tryptophan hydroxylase
IECs: Intestinal epithelial cells
TYK2: Tyrosine kinase 2
CXCR2: C-X-C motif chemokine receptor 2
SOCS3: Cytokine signaling 3
IKK: IκB kinase
JNK: c-Jun N-terminal kinases
IL-22BP: Interleukin-22-binding protein
IL-22R1: Interleukin-22 receptor subunit 1
FXR: Farnesoid X receptor
FMO3: Flavin-containing monooxygenase 3
FFAR2: Free fatty acid receptor 2
AMPs: Antimicrobial peptides
MHC II: Major histocompatibility complex class II
NIK: NF-kB-inducing kinase
PIAS3: Protein inhibitor of activated STAT3
CVD: Cardiovascular disease
DMB: 3, 3-dimethyl-1-butanol
IMC: Iodomethylcholine
FCAR: R-N-fluoromethylcarnitine
Gly-β-MCA: Glycine-β-muricholic acid
NRP1: Neuropilin-1
uSTAT3: Unphosphorylated STAT3
MLCK: Myosin light-chain kinase
uNF-κB: Unphosphorylated NF-κB
NEC: Neonatal necrotizing enterocolitis
